# Anti-HIV Potential of *Origanum vulgare* Compounds Targeting Viral Reverse Transcriptase with High Binding and Stability Validated by Machine Learning

**DOI:** 10.3390/v18091010

**Published:** 2026-09-13

**Authors:** Leena Hussein Bajrai, Reem Ghazali

**Affiliations:** 1 Biochemistry Department, Faculty of Science, King Abdulaziz University, Jeddah 21362, Saudi Arabia; 2 Special Infectious Agents Unit-BSL3, King Fahd Medical Research Centre, King Abdulaziz University, Jeddah 21362, Saudi Arabia; 3 Department of Clinical Biochemistry, Faculty of Medicine, King Abdulaziz University, Jeddah 21362, Saudi Arabia; rghazaly@kau.edu.sa; 4 Food, Nutrition and Lifestyle Unit, King Fahd Medical Research Centre, King Abdulaziz University, Jeddah 21362, Saudi Arabia

**Keywords:** HIV-1 reverse transcriptase, *Origanum vulgare*, phytochemicals, machine learning QSAR

## Abstract

Human immunodeficiency virus (HIV) is one of the viruses that has co-evolved within human populations for a considerable time. With the evolution of new drug-resistant HIV strains, the necessity to discover novel drugs has become an issue of great concern, especially those that have increased binding affinities and inhibitory activity towards RT enzymes. This study used an in silico approach to identify potential HIV-RT inhibitory candidates from *Origanum vulgare* (oregano). An initial in silico screening of approximately 820 compounds was conducted, and based on molecular docking-derived binding energy scores, four compounds (IMPHY000687, IMPHY007084, IMPHY004619, and IMPHY012021), with docking scores of −9.42, −9.32, −9.24, and −9.23 kcal/mol, respectively, were selected as the top-ranked phytocompounds and were subsequently validated using multiple computational approaches. These compounds were geometrically optimized using quantum-chemical calculations, and detailed interaction analyses were performed using a redocking procedure. Reproducibility of the dynamic behavior was evaluated by carrying out independent replica molecular dynamics simulations for 300 ns each for all complexes. The ligand-dependent conformational dynamics were identified using RMSD and RMSF analyses, along with variations in positional changes during simulation times. PCA and FEL analyses helped in identifying the conformations sampled by the system under study. In addition, QM/MM calculations provided complementary information on the electronic characteristics of the individual protein–ligand systems. Machine learning-based quantitative structure–activity relationship (QSAR) prediction of experimentally validated HIV-RT inhibitors was applied to predict inhibitory potency, yielding predicted pIC50 values for the selected phytochemicals compared with the reference molecule. All in all, comprehensive computational analyses have ranked these phytochemicals as HIV-RT inhibitors that need further experimental verification.

## 1. Introduction

Despite substantial advancements in the treatment and prevention strategies over the last four decades, human immunodeficiency virus (HIV) remains a significant public health concern. According to the World Health Organization (WHO) report published at the end of 2024, approximately 40.8 million people worldwide suffer from HIV, with approximately 1.3 million new cases reported during the same year [1]. Moreover, the mortality rate remains high, with 630,000 deaths worldwide, emphasizing the persistent burden of disease. Even with the considerable expansion of antiretroviral therapy, only 87% of HIV patients are aware of their infection status, while 77% patients are undergoing treatment and approximately 73% of HIV patients have recovered through viral suppression. Most low- and middle-income countries are still affected by this epidemic [1]. The situation in these countries indicates that HIV transmission and disease spread still need to be controlled. Therefore, the need for continuous therapeutic innovation persists.

The replication mechanism of HIV depends mainly on the activity of reverse transcriptase (RT), a multifunctional enzyme that aids in the conversion of the viral single-stranded RNA genome into double-stranded DNA. This step, also known as reverse transcription, is important for the integration of viral DNA into the host genome, followed by viral replication [2]. Therefore, RT has been the central target in antiretroviral drug development. Several HIV-RT inhibitors have been identified and clinically approved to enhance treatment strategies [3]. Currently, the clinically approved HIV-RT inhibitors are classified into two groups: nucleoside (NRT) and non-nucleoside RT inhibitors (NNRT). Nucleoside RT inhibitors, such as zidovudine and lamivudine, act as nucleotide analogs that stop the DNA elongation process, whereas non-nucleotide RT inhibitors, for example, efavirenz and nevirapine, bind to the allosteric pocket of the enzyme and inhibit enzyme activity through conformational modulation [4]. Although these inhibitors are highly effective antiretroviral therapeutic agents, their long-term usage leads to toxicity and the emergence of drug resistance [5].

Treatment failure due to resistance mechanisms has encouraged the identification of new HIV-RT inhibitors to overcome the limitations of existing inhibitors [6]. For instance, RT translocation inhibitors mainly aim to disrupt the physical movement of the enzyme along the nucleic acid template during DNA synthesis. The inhibition of enzyme translocation rather than blocking replication or allosteric signalling offers a promising strategy to restrain resistant strains [7,8]. MK8527 is the best example of an RT translocation inhibitor because of its acceptable pharmacological properties and ability to support extended-duration dosing. The development of translocation inhibitors also aids in reducing dosage frequency and improving patient adherence to HIV treatment and prevention [9]. Recent advancements in computational drug discovery have significantly accelerated the identification of novel HIV-1 reverse transcriptase (HIV-RT) inhibitors. In silico approaches, including quantitative structure–activity relationship (QSAR) modelling, molecular docking, molecular dynamics (MD) simulations, and binding free energy calculations, have been widely employed to screen and optimize potential inhibitors with improved efficacy and stability [10].

Despite these therapeutic advancements, only a few chemically diverse RT translocation inhibitors have been developed to date, highlighting the need for new molecular compounds with inhibitory functions [11]. Natural compounds from various biological sources have significantly contributed to antiviral drug discovery because of their structural and biological importance [12]. Furthermore, medicinal plants have been employed as sources of secondary metabolites, which act as significant antiviral drugs [13]. The phytochemicals of *Origanum vulgare* include phenolic acids, flavonoids, and terpenoids. They possess antimicrobial, antioxidant, and antiviral activities [14]. However, the inhibitory effect of phytochemicals of oregano against HIV-RT, especially via a translocation mechanism, has not been sufficiently researched. Modern computational approaches in drug discovery allow for a rapid and economically efficient screening of natural substances against target proteins of viruses [15]. The computational methods can assess in detail the interactions of ligands with binding sites regarding their affinity, stability, and energetics under physiological conditions [16]. This approach is useful when studying the dynamics of the protein HIV-1 reverse transcriptase because of the protein flexibility in the process of ligand binding and stabilization.

For this current investigation, a detailed computational approach was adopted to screen potential HIV-1 reverse transcriptase inhibitors from *Origanum vulgare* through a computational pipeline involving molecular docking, 300 ns of molecular dynamics simulations to predict the stability and dynamic nature of the protein–ligand complex, density functional theory (DFT), quantum mechanics/molecular mechanics (QM/MM) analysis, and QSAR modeling based on machine learning algorithms. The multi-step approach provides complementary information to predict ligand binding and stability and adds to the robustness of computational prediction. This combined computational methodology helps achieve an understanding of the interactions between the phytochemicals of oregano at the molecular level within the binding site of the MK-8527 binding pocket of the p66 subunit of HIV-RT without trying to replicate the entire process of HIV-RT translocation.

## 2. Methodology

### 2.1. Selection and Preparation of HIV Reverse-Transcriptase Structures

The crystal structure of HIV-RT (Protein Data Bank ID 9DM9) was obtained from the Protein Data Bank [9,17,18]. In the current study, the pre-catalytic complex structure of HIV-RT was chosen, which was crystallized by the X-ray crystallography technique with a resolution of 2.37 Å. This structure is based on the HIV-1 HXB2 isolate, which provides insight into its biological importance in reverse transcription and translocation mechanisms. The selected protein was prepared using UCSF Chimera version 1.13.1 [19]. The p66 catalytic subunit (Chain A) was selected for docking and molecular dynamics simulations because it contains the experimentally characterized MK-8527 binding pocket used in the present study. The p51 subunit, nucleic acid, metal ions, and other heteroatoms were removed during receptor preparation to facilitate structure-based ligand screening. Accordingly, the computational analyses were designed to evaluate ligand interactions within the validated binding pocket rather than to reproduce the complete HIV-RT translocation mechanism. Moreover, the crystallographic water molecules were eliminated while retaining the overall structural integrity of the protein. Missing atoms and residues were fixed in the required regions, and hydrogen atoms were added to assign the correct protonation states under physiological conditions. The final prepared protein structure was then subjected to energy minimization to reduce steric clashes and geometric optimization.

### 2.2. Compilation and Preparation of Origanum vulgare Phytochemical Library

The phytochemical compounds of the Oregano plant (*Origanum vulgare*) were collected from the IMPAT (Indian Medicinal Plants Phytochemistry and Therapeutics) database (https://cb.imsc.res.in/imppat/, accessed on 5 December 2025), a curated repository of experimentally reported bioactive compounds associated with medicinal plants. [20]. The database was systematically queried using the keyword “*Origanum vulgare*” to retrieve all reported phytocompounds. All reported phytocompounds associated with *O. vulgare* were initially downloaded and compiled into a unified ligand library. Redundant entries were identified and removed based on compound name, ID, and canonical SMILES notation. Compounds lacking structural information or having incomplete chemical characterization were excluded from further analysis. Compounds with ambiguous stereochemistry, salts, or inorganic components were also excluded. After multi-step curation and filtration, 820 unique and structurally validated phytochemicals were finalized and used for subsequent virtual screening in SDF format and imported into Progenesis SDF Studio (https://www.nonlinear.com/progenesis/sdf-studio/, accessed on 5 December 2025) for ligand library preparation and standardization. The construction of the ligand library was in accordance with the phytochemical database.

### 2.3. Structure-Based Virtual Screening

The phytochemical database was screened against HIV-RT through the use of the MTi Open Screen server (https://bioserv.rpbs.univ-paris-diderot.fr/services/MTiOpenScreen/, accessed on 5 December 2025) [21]. The structure of the prepared HIV-RT protein, along with the phytochemical database, was uploaded to the web server, and the docking grid was set up using the coordinates of the native ligand MK 8527 (x = −41.1, y = −1.38, z = −26.13) with grid dimensions of 20 Å. In this way, all the compounds would be docked in the same region where the native ligand is docked. Based on docking scores during the screening process, those compounds with favorable energetics were chosen for further computational studies.

### 2.4. ADMET Analysis

The ADMET analysis of the top compounds was carried out based on the pharmacokinetic and toxicological features using ADMET Lab 2.0—a powerful and reliable platform [22]. Such software provides extensive information about the ADMET properties of the compounds in question. In particular, the following parameters were considered: gastrointestinal absorption, BBB permeability, Vd, PPB, metabolic stability, and potential interactions with cytochrome P450 enzymes. Moreover, several toxicity-related features were assessed, namely, hepatotoxicity, cardiotoxicity, and the Ames test. The ADMET Lab 2.0 analysis results were highly valuable for selecting promising drug-like molecules and assessing the possibility of their further use as potential candidates for therapeutic agents.

### 2.5. Quantum Chemical Optimization and Electronic Property Analysis

To gain a better understanding of the electronic behaviour and reactivity of the selected phytochemical compounds, quantum chemical calculations were performed using DFT. The 3D geometries of the selected ligands were extracted from the SDF files using RDKit [23,24,25]. This ensured the preservation of hydrogen atoms and the spatial arrangement of the selected compounds. For the comparative evaluation, the native inhibitor MK 8527 molecule was included as a reference molecule and subjected to DFT calculations. DFT calculations were performed using the PySCF software version 2.14.0 [26,27]. The geometry optimisation of the selected compounds and the reference molecule was carried out using the B3LYP exchange-correlation functional combined with the cc pVDZ basis set [28]. The optimisation procedure aided in the relaxation of the molecular geometry without imposing system constraints, which provides an accurate evaluation of electronic distribution. Additionally, the key electronic descriptors were obtained from the converged wave function. The energies of the highest occupied molecular orbital (HOMO) and lowest unoccupied molecular orbital (LUMO) were calculated to measure the molecular stability and chemical reactivity of the selected compounds. Orbital density maps were also generated to visually examine the electron distribution in the compounds.

### 2.6. Molecular Redocking and Interaction Assessment

To further analyze the reliability of the optimized compounds, a re-docking analysis was performed using the AutoDock Vina version 2.0 plugin integrated into UCSF Chimera [19,29]. The docking grid used during the screening process was retained for all redocking experiments. Before conducting re-docking, to ensure that the binding pose was accurately represented, the protocol was validated by re-docking the native ligand MK-8527 into its MK-8527 binding pocket to confirm the accuracy of the binding pose. Specifically, the co-crystallized ligand of HIV-1 reverse transcriptase, MK-8527, is a known inhibitor of HIV-1 reverse transcriptase [9]. MK-8527 potential effect in adult human is also well known in recent study [30]. The comparable binding affinities and interaction profiles observed between the reference inhibitor MK-8527 and the top-ranked phytochemicals further support the robustness and reliability of the docking parameters. The redocked pose was superimposed onto the crystallographic pose, and the root mean square deviation (RMSD) was calculated using PyMOL version 3.1.0 [31]. An RMSD value of 0.6 Å was obtained, confirming that the redocked pose and experimental poses have identical orientations. Following validation, the selected phytochemicals were re-docked into the MK-8527 binding pocket, and the residue-level interaction patterns of each complex were examined using Discovery Studio software version 24.1 to assess their binding stability [32].

### 2.7. Molecular Dynamics Simulation of Protein–Ligand Complexes

MD simulation studies were performed in order to determine the stability, conformational dynamics, and interaction stabilization ability of HIV-RT in complex with certain phytochemicals and a control ligand. MD simulations were performed for each protein–ligand complex for a duration of 300 ns. To improve the robustness and reproducibility of the results, independent replicate simulations were carried out using different initial velocity distributions. Each system was energy minimized, equilibrated, and subjected to production runs under periodic boundary conditions. Additionally, the apo form of HIV-RT was simulated under identical conditions. The simulations were performed using the AMBER software version 2.0.0 package [33]. The protein atoms were parameterized using the ff14SB force field, whereas the selected compounds were treated using the General Amber Force Field (GAFF) [34]. The Ligand topologies and parameters were generated using the Antechamber module of AmberTools, and partial atomic charges were assigned using the AM1-BCC charge model. The ligand structures used for parameterization retained the protonation and tautomeric states present in the prepared input structures, and no additional protonation or tautomer optimization was performed before molecular dynamics simulations. The LEaP module aided in system preparation for the simulation of both apo and ligand-bound proteins. The TIP3P water module was used as the water model for solvating each system using a truncated octahedral box with a solute-boundary distance of 10 Å [35]. The system was neutralized by adding the required amount of sodium counterions. Moreover, the systems were energy-minimized before the simulation to remove unfavorable interactions and relax the solvent molecules surrounding the solute. The temperature of the minimized system was regulated from 0 K to 300 K over 100 ps under constant-volume conditions using a Langevin thermostat with a collision frequency of 2 ps^−1^. Subsequently, the system density and volume were stabilized using the Berendsen barostat at 1 atm [36]. The simulations were run for 300 ns for the apo protein and each protein–ligand complex under constant temperature and pressure. A 2 fs time step was employed, and the major covalent bonds were restricted by the SHAKE algorithm, and the long-range electrostatic interactions were bounded using the Particle Mesh Ewald method, with a non-bonded interaction cutoff of 8 Å [37,38]. The final trajectory was analyzed to evaluate structural stability and conformational behavior by calculating RMSD, Root mean square fluctuation (RMSF) to evaluate the residual level flexibility and identify regions of major deviations. Moreover, hydrogen bonds formed in the simulated complexes were monitored to assess interaction persistence under dynamic conditions. Furthermore, the final pose was extracted from the trajectory to observe the residue-level contacts and compare them with docking-based interaction patterns to validate the ligand-binding pattern. Two separate MD simulations were run for each protein–ligand complex. This included an MD simulation and an independent replicate. Both simulations were run for a total of 300 nanoseconds each using different velocities.

### 2.8. Conformational Dynamics Analysis Using Principal Component Analysis and Free Energy Landscapes

PCA was performed using the GeoMeasures plugin to investigate the conformational motion of HIV-RT and the dynamic nature of phytochemical compound [39]. This analysis enables the construction of covariance matrices and the extraction of eigenvalues and eigenvectors displaying dominant modes of motion. The principal components (PCs) with the highest eigenvalues were considered most relevant as they account for the maximum variance observed throughout the trajectories. The first two PCs were selected for the reaction coordinates to map the free energy landscape (FEL) of the complexes. Through FEL analysis, the distribution of the conformational states sampled during the simulations. The FEL maps were plotted using GeoMeasures and visualised in PyMOL [31]. Further, the global energy minima structures were extracted from each FEL profile, and the conformers were superimposed for the comparative examination of structures. The examination assesses the conformational changes and stabilisation of each ligand within the binding region.

### 2.9. Quantum Mechanics and Molecular Mechanics Based Calculations

To understand the protein–ligand interaction of each complex beyond the classical force field, quantum mechanics/molecular mechanics (QM/MM) calculations were conducted using the PySCF platform [26,27,40]. This technique captures electronic level interaction that are not counted during the MD simulation. For each phytochemical, the lowest energy conformer was extracted from the simulation trajectory and used as the starting point for QM/MM analysis. A double-layer partitioning method was used, wherein the bound ligand formed the QM region and the surrounding amino acids in the MK-8527 binding pocket were treated at the MM level. The QM region was determined using the B3LYP exchange correlation functional in combination with the def2 SVP basis set, which aids in balancing the accuracy and computational cost of the compounds [28]. Further, an electrostatic embedding scheme was applied, allowing the electrostatic field of the MM region to influence the QM region. This helped in the inclusion of polarization effects arising from the protein environment, thereby stabilizing the ligand within the MK-8527 binding pocket. The QM/MM calculation was applied to gain additional insight into the electronic properties of the protein–ligand complexes within the binding site of MK-8527, while the ligands were not ranked based on their binding affinities.

### 2.10. Machine Learning-Based QSAR Modeling for HIV-RT Inhibition

In the present study, the machine learning-based Quantitative Structure–Activity Relationship approach was employed to predict the inhibitory activity of the phytochemical compounds derived from oregano against the HIV-1 reverse transcriptase enzyme [41]. The experimentally validated set of 165 compounds with known IC_50_ values against the HIV-1 RT enzyme was downloaded from the ChEMBL database [42]. The IC_50_ values were converted to the corresponding pIC_50_ values using the negative logarithm transformation. The structural representation of the compounds was performed through the dual descriptor approach. The RDKit library was used to calculate the physicochemical descriptors, while the structural and topological descriptors were generated through the PaDEL Descriptor tool version 2.21 [24,43]. The generated descriptors provided comprehensive coverage of the relevant descriptors required for the inhibitory activity. The descriptors with low variance were eliminated, while the high correlation between the descriptors was filtered out through the Pearson correlation coefficient filter implemented through the scikit-learn library. The filtered dataset was then divided into the training set and the testing set in an 80:20 ratio. The missing values were imputed with the mean values, while the normalization of the descriptors was performed through the StandardScaler. A wide range of machine learning regression techniques were employed, including Random Forest, Extra Trees, Gradient Boosting, XGBoost, CatBoost, Support Vector Regression, Ridge, Lasso, Elastic Net, K-Nearest Neighbors, Multilayer Perceptron Regression, Decision Trees, AdaBoost, Bagging, Histogram-based Gradient Boosting, etc. The hyperparameters were optimized through the randomized grid search algorithm with cross-validation. Assessment of the possibility of overfitting was done by examining the performance of the model in both the training set and the validation set. Assessment of model performance was done based on the values of R^2^, RMSE, and MAE. Apart from that, five-fold cross-validation was also used during model assessment. The performance metrics were visualized through bar plots, residual plots, and correlation plots generated through the seaborn and matplotlib libraries. The predicted pIC_50_ values were generated for the phytochemical compounds derived from oregano and the known HIV-RT inhibitor through the best-performing machine learning regression models.

## 3. Results

### 3.1. Structure-Based Virtual Screening

**Figure 1:**
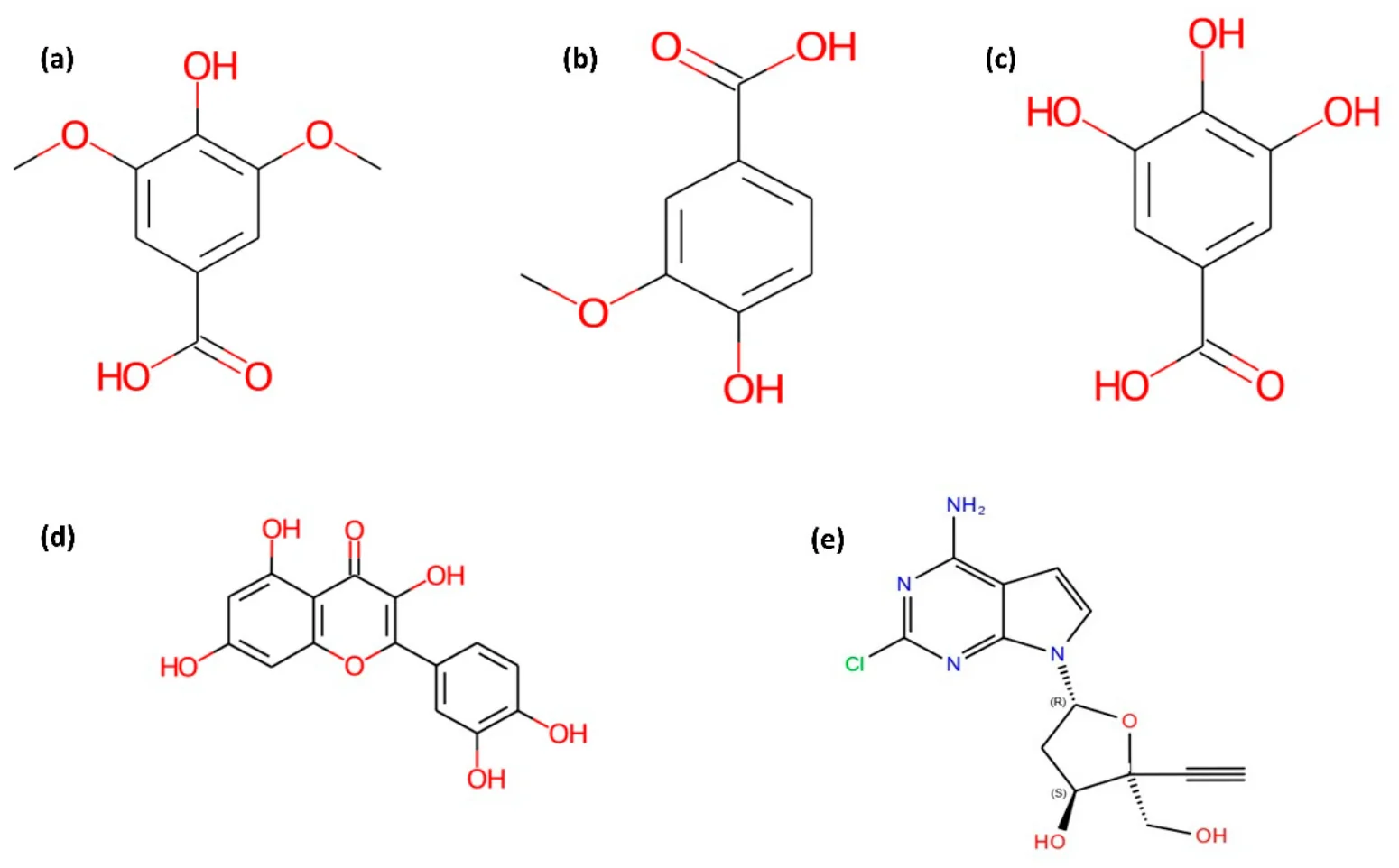
Two-dimensional structures of phytocompounds identified from the PubChem database. (**a**) IMPHY000687; (**b**) IMPHY007084; (**c**) IMPHY012021; (**d**) IMPHY004619; (**e**) reference inhibitor MK-8527.

Virtual screening of the phytochemical library of *Origanum vulgare* that consists of 820 phytochemicals, was done using the HIV-RT protein. After virtual screening, a total of 353 phytochemicals provided valid docking values, whose score values are from −1.01 to −9.42 kcal/mol (Supplementary Table S1). Four phytochemicals were selected as top-ranked computational candidates according to the ranking of binding energies. IMPTHY000687 showed the maximum binding energy, with a docking score of −9.42 Kcal/mol, followed by IMPHY007084 (−9.32 Kcal/mol), IMPHY004619 (−9.24 Kcal/mol), and IMPHY012021 (−9.23 Kcal/mol). Selected phytochemicals were subjected to DFT optimization and electronic property calculations due to their superior docking scores. Figure 1 depicts the structure of the selected phytochemicals.

### 3.2. ADMET Analysis

The above-mentioned phytochemicals (IMPHY000687, IMPHY007084, IMPHY004619, and IMPHY012021) were further evaluated through ADMET analysis for evaluating their pharmacokinetics and safety properties. In general, all four compounds possessed good in silico drug-likeness and pharmacokinetic properties. However, some differences in the absorption, metabolism, and toxicity predictions for these molecules should be considered, as the use of both ADMET and docking and molecular dynamics is crucial in lead molecule selection.

According to the ADMET profile of the selected drugs (IMPHY000687, IMPHY007084, IMPHY004619, and IMPHY012021), they meet Lipinski’s Rule of Five, which suggests good druggability properties and oral bioavailability of such candidates, as shown in Table 1. Predicted probabilities of HIA+ varied between 0.013 and 0.085 for the chosen phytochemicals. In accordance with the ADMETlab 2.0 categorization, these relatively low probabilities (<0.3) mean a small chance of poor intestinal absorption and predict favorable intestinal absorption for all four phytochemicals. According to the BBB penetration prediction analysis, IMPHY000687 and IMPHY007084 showed moderate BBB permeation probability values of 0.457 and 0.439, respectively. Meanwhile, IMPHY004619 had the lowest BBB permeation value, 0.008. The NP-likeness indices ranged between 0.524 and 1.701, with IMPHY004619 showing maximum similarity to natural products. The volumes of distribution (VD) were between 0.358 and 0.579, suggesting moderate tissue distribution, with IMPHY004619 showing comparative distribution. Regarding interaction with metabolism, there was a high likelihood (0.943) of IMPHY004619 inhibiting CYP3A4, while the other compounds had minimal potential to do so. There was a prediction of higher mutagenicity probability (0.657) in AMES toxicity test for IMPHY004619, while the rest of the compounds were less toxic. Overall, IMPHY000687, IMPHY007084, and IMPHY012021 had comparatively better predicted pharmacokinetics and safety parameters. On the other hand, although IMPHY004619 showed good binding properties and stability, due to its predicted inhibition of CYP3A4 and high AMES mutagenicity probability, it cannot be considered a candidate until further optimized.

### 3.3. Density Functional Theory-Based Electronic Structure Analysis

**Figure 2:**
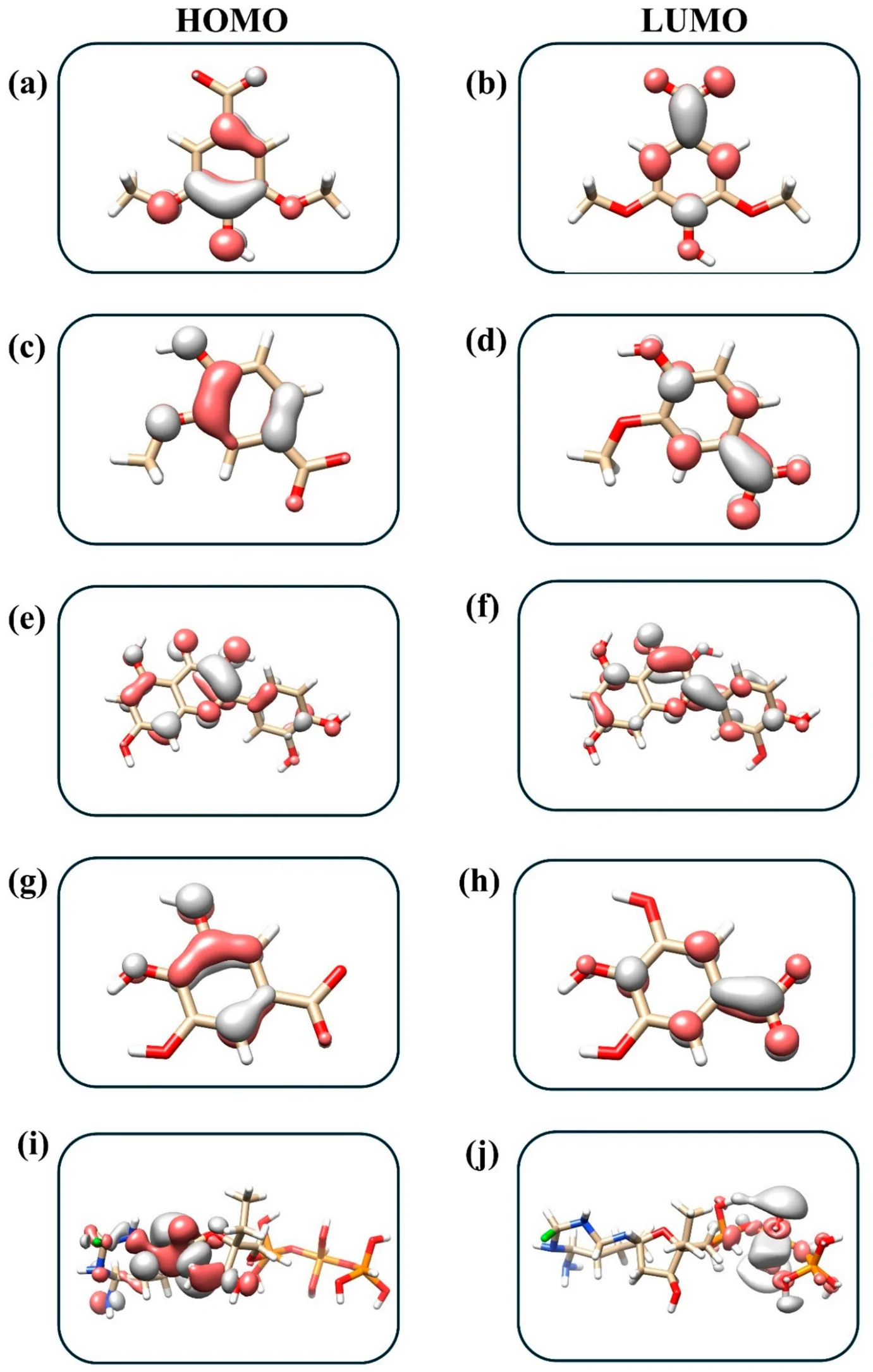
HOMO–LUMO distribution of selected compounds: (**a**,**b**) IMPHY000687, (**c**,**d**) IMPHY007084, (**e**,**f**) IMPHY004619 and (**g**,**h**) IMPHY012021, and (**i**,**j**) reference inhibitor.

Electronic features of the highest-ranking phytochemicals and the reference inhibitor were evaluated through density functional theory (DFT) computations. Frontier molecular orbitals analysis was carried out to study molecular stability, reactivity, and electronic features of the mentioned compounds. For compound IMPHY000687, the HOMO and LUMO energies are −6.18 eV and −1.66 eV, respectively, giving a HOMO-LUMO gap of 4.52 eV. The orbital distributions are highly localized on heteroatoms, reflecting areas of possible electron donation and acceptance. For phytochemical IMPHY007084, the HOMO energy equals −6.40 eV, the LUMO energy equals −1.70 eV, giving a HOMO-LUMO gap value of 4.70 eV. The orbital density is distributed across multiple functional groups, indicating several reactive centers in the molecule. In the case of IMPHY004619, the HOMO energy level is −5.59 eV and the LUMO energy level is −1.56 eV, giving the HOMO–LUMO gap of 4.03 eV. Such a small energy gap demonstrates high reactivity of selected phytochemicals from the electronic perspective. The HOMO energy level in IMPHY012021 is −6.42 eV, while the LUMO energy level is −1.69 eV. Consequently, the HOMO-LUMO gap is 4.73 eV. Similarly high electronic characteristics can be observed in other shortlisted phytochemicals. The HOMO energy level of the reference compound is −5.52 eV, the LUMO energy level is −0.63 eV, and consequently the HOMO–LUMO gap is 4.89 eV. Importantly, molecular stability and chemical reactivity were studied using the HOMO–LUMO energy gap. The HOMO-LUMO energy distribution in phytochemicals is provided in Figure 2.

### 3.4. Redocking Validation and Interaction Analysis of Selected Phytochemicals

**Figure 3:**
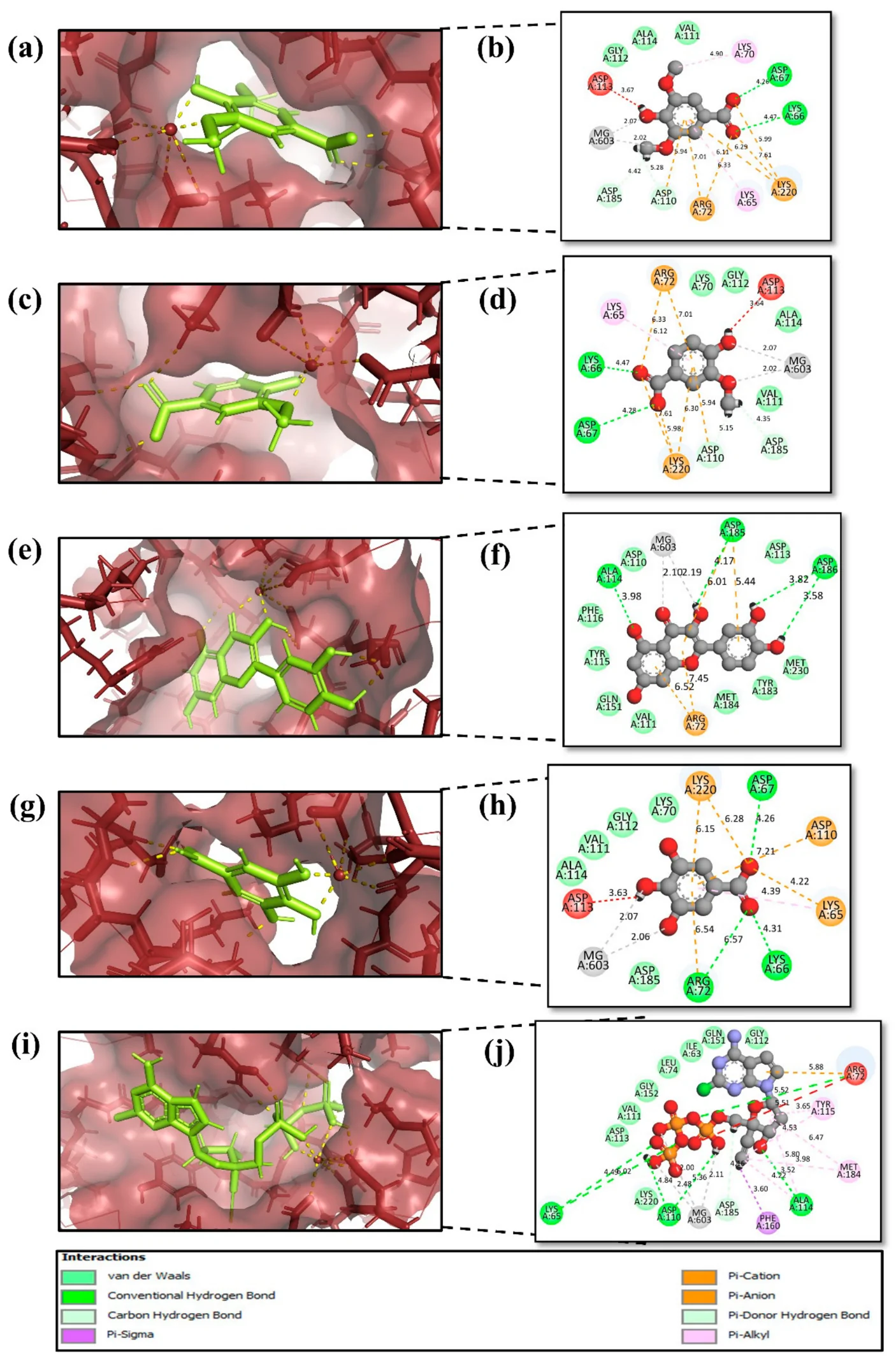
Combined two-dimensional and three-dimensional interaction representations of HIV-RT with (**a**,**b**) IMPHY000687, (**c**,**d**) IMPHY007084, (**e**,**f**) IMPHY004619 and (**g**,**h**) IMPHY012021, and (**i**,**j**) reference inhibitor.

Re-docking analysis was carried out to ascertain the binding affinity of the selected phytochemicals. The re-docking energies of the phytochemicals IMPHY000687, IMPHY007084, IMPHY004619, and IMPHY012021 are −9.28, −9.34, −9.24, and −9.18 kcal/mol, respectively, compared to the reference compound’s energy of −9.06 kcal/mol. From these findings, it is clear that the selected phytochemicals have higher binding affinity than the reference compound towards the target protein. Re-docking analysis was further carried out to find out how the selected ligands stabilize within the MK-8527 binding pocket of the target protein by showing the interactions of the complexes formed between the HIV-RT and the phytochemicals. The complete interaction profile has been summarized in Table 2, while the 2D and 3D interaction diagrams are shown in Figure 3. The residue-level interactions were analyzed using Discovery Studio Visualizer. For quantitative analysis, only conventional hydrogen bonds with donor–acceptor distances ≤ 4.5 Å were considered and reported in the interaction table, whereas other non-covalent interactions, including hydrophobic and π-mediated interactions, were classified according to the interaction definitions provided by the software. The HIV-RT-IMPHY000687 complex formed hydrogen bonds with Lys66 and Asp67, residues that play an important role in anchoring the ligand within the MK-8527 binding pocket. A similar hydrogen bonding pattern was observed in HIV-RT-IMPHY007084 complex and HIV-RT-IMPHY012021 complex. In both these complexes, Lys66, and Asp67, participated in a hydrogen bond, indicating the significance of these residues in ligand stabilisation. However, in HIV-RT-IMPHY004619, the complex exhibited hydrogen bond interaction involving Ala114 and Asp185, with Asp186 forming two hydrogen bonds, indicating an altered but still stabilising polar interaction. The reference molecule showed multiple hydrogen bonds Lys65 (five interactions), Arg72 (two interactions), Asp110 (two interactions), and Ala114, displaying strong polar interactions within the complex. In addition to hydrogen bonds, π–π stacking and π-associated interactions significantly contributed to ligand stabilisation. IMPHY000687 engaged in π interactions with Lys65, Lys70, Arg72, Asp110, and Lys220, providing electrostatic and aromatic interactions. Similar π-interaction patterns were observed for IMPHY007084 and IMPHY012021, involving Lys65, Arg72, Asp110, and Lys220. For IMPHY004619, π-associated interactions were formed by Arg72 (two interactions) and Asp185 (two interactions). The reference molecule displayed multiple π interactions involving Arg72, Ala114 (two interactions), Tyr115 (two interactions), and Met184 (two interactions), further highlighting the importance of aromatic and charged residues in binding stabilization. Hydrophobic interactions made up the biggest number of interactions in all the complexes and involved several residues within the MK-8527 binding pocket. Hydrophobic interactions were dominant in all the complexes, proving their importance in the stabilization of protein–ligand interactions.

To draw clinical relevance, the identified molecules were analyzed in parallel with frequently used HIV RT inhibitors such as delavirdine, efavirenz, and nevirapine [2,44]. The selected compounds (IMPHY000687, IMPHY007084, IMPHY004619, and IMPHY012021) had docking scores equal to or higher than those of the reference inhibitors (for instance, efavirenz −6.5 kcal/mol, nevirapine −7.2 kcal/mol, delavirdine −8.6 kcal/mol). Interaction analysis has shown that all selected phytocompounds share key binding sites with the control inhibitor (such as Lys65, Arg72, Asp110, and Ala114), indicating conserved binding in the MK-8527 binding pocket. In particular, IMPHY004619 was found to have a broad spectrum of hydrophobic and π-interactions with binding-pocket residues (Asp185 and Tyr115), suggesting its superior stability compared with the other molecules. A detailed 2D interaction profile of the reference inhibitors, delavirdine, efavirenz, and nevirapine, is provided in Supplementary Figure S9.

### 3.5. Molecular Dynamics Simulation Analysis

#### 3.5.1. RMSD Analysis

**Figure 4:**
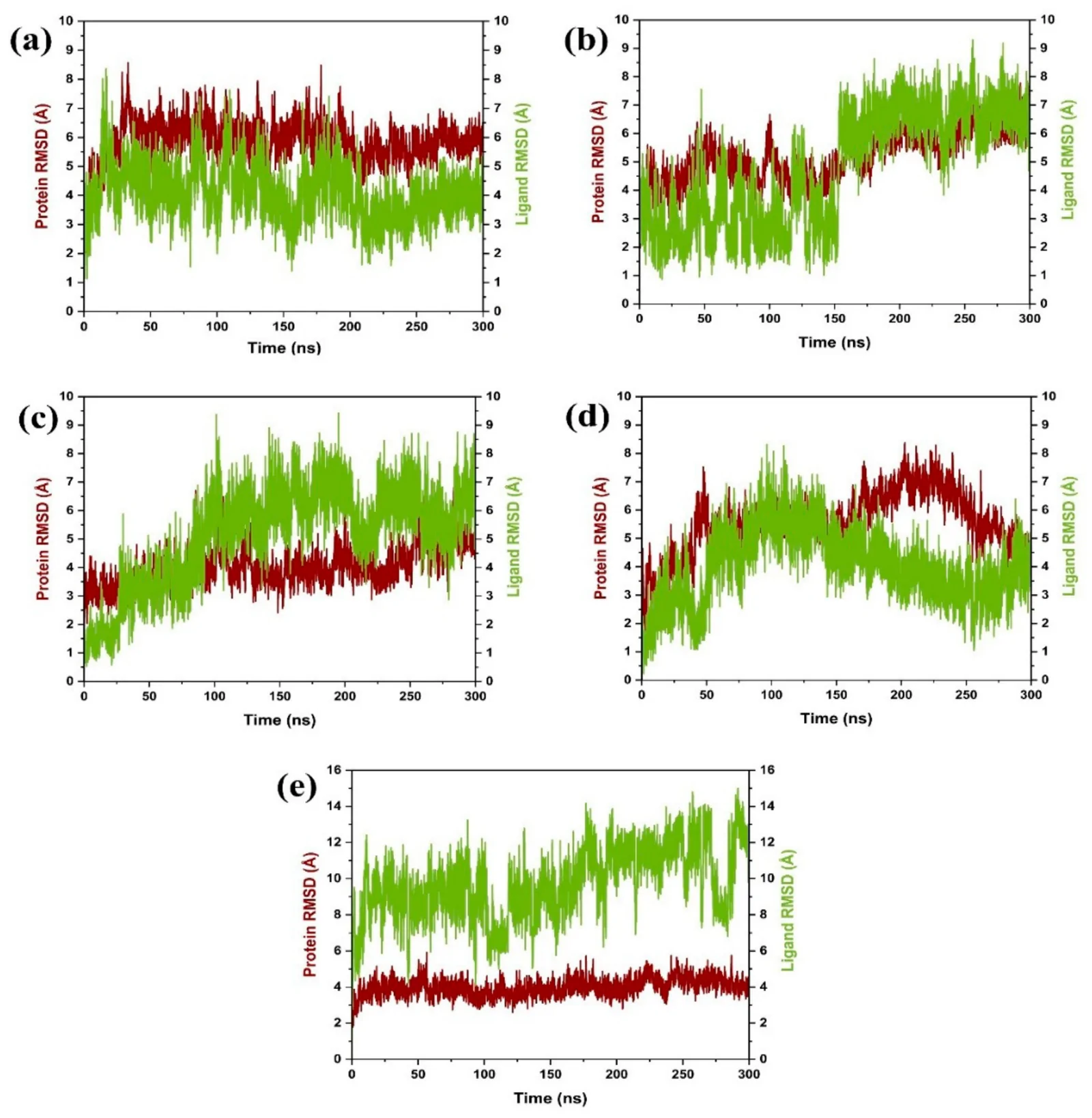
RMSD profiles of HIV-RT complexes with (**a**) IMPHY000687, (**b**) IMPHY007084, (**c**) IMPHY004619, (**d**) IMPHY012021, and (**e**) reference inhibitor.

The foundation of this research included RMSD analysis, which aimed at understanding the structural stability and conformational dynamics of the HIV-RT protein in the presence of the aforementioned phytochemicals. In the case of HIV-IMPHY000687, the RMSD of the protein initially reached values of 7–8 Å. Meanwhile, the ligand RMSD showed larger fluctuations (7–8 Å) initially, but then reduced and stabilized around 3.5–5.5 Å within 150 ns. Overall, this indicates partial mobility of the ligands with partial stability within the binding pocket. Thus, there is some positional change in the ligand-protein interaction and not a fixed one (Figure 4a). For the HIV-IMPHY007084 complex, the protein RMSD peaked at 6 Å; however, after 150 ns, the protein deviated around 7 to 8 Å till the end of the simulation. A similar scenario was observed in the case of IMPHY007084 RMSD; till 150 ns, the ligand deviated to 5.5 Å, followed by a gradual increase after 150 ns to 6–8 Å till the later stages of the simulation. This behaviour indicates significant positional displacement of the ligand relative to its initial docked conformation, suggesting a dynamic binding mode with possible repositioning rather than stable anchoring (Figure 4b). The HIV-RT-IMPHY004619 complex, the protein RMSD remained in the range of 5–6 Å; however, significant peaks were observed that reached up to 7 Å at various intervals of simulation. This indicates that the protein displayed overall structural stability. The ligand RMSD showed a gradual rise from 3 Å during the initial simulation stage to 6.5 to 8.5 Å towards the end of the simulation, with peaks at multiple intervals that reached up to 9 Å. A prolonged binding-site displacement suggests continuous repositioning of the ligand inside or adjacent to the MK-8527 binding pocket, which is an indication of no stable and restricted binding (Figure 4c). In the HIV-IMPHY012021 complex, the RMSD of the protein increased up to 7.5 Å during the first stage of the simulation and decreased further to stabilize at around 5 Å in the final stage of the simulation, showing equilibrium and stability of the protein structure. On the other hand, the ligand’s RMSD showed a steady increase up to 8 Å starting from 4 Å in the first 100 ns and decreased to around 3–5 Å after the simulation period. This reduction in RMSD during the later stages indicates that the ligand adopts a relatively stable conformation after initial repositioning, suggesting partial retention within the binding region (Figure 4d). Protein RMSD in the reference complex was quite consistent and close to 4 Å for the entire duration of the simulation, showing low deviation from the protein structure. On the contrary, the reference ligand had an RMSD value of 10–14 Å compared to its original position after docking, showing significant displacement of the ligand within the first trajectory. Such a high value of RMSD implies that there is a lot of displacement of the ligand within the binding site; however, a high value of RMSD does not confirm dissociation of the ligand (Figure 4e).

The RMSD of the apo protein increased from 4 Å to 6.5 Å over the course of the simulation, indicating inherent structural flexibility of the protein in the absence of ligand binding. This behaviour suggests that ligand binding may influence the conformational dynamics of the protein (Supplementary Figure S1). Moreover, the observed higher RMSD values may arise from ligand flexibility, initial docking pose adjustments, and conformational adaptation of the binding pocket.

To evaluate the reproducibility of molecular dynamics simulations, independent replicas were generated from different initial seeds. The time series of RMSD values from the replica trajectories demonstrated similar patterns to those obtained in the original simulations about equilibration of protein and ligand mobility; the RMSD values for ligand positions in the replicas were found to be bounded and varied in the range of 3–6.5 Å over the course of the simulation for all phytochemical complexes. These data indicate that the ligands are actively moving throughout the simulation but are not moving continuously or progressively from the binding site during these periods of active motion. In some instances, transient stabilization and subsequent re-mobility were observed, which suggests that there is a flexible, adaptable mechanism of interaction between ligands and proteins. Conversely, the RMSD values for reference ligands in the replicas were consistently greater than those for the primary simulation and were found to be in the range of 6–10 Å without any sustained stabilization. Thus, this study supports the results of the initial simulation and suggests that there is a significant amount of positional drift and reduced stabilization of interaction compared to phytochemical ligands; however, the replicas were not used to make statistical inferences, but rather to provide additional qualitative evidence of the reproducibility of the trends observed in the initial experiments. The RMSD profiles of the replica simulations for all complexes are presented in Supplementary Figure S2, showing comparable fluctuation patterns across independent trajectories.

To quantitatively assess the reproducibility of the MD simulations, independent replicate simulations were performed using different initial velocity distributions. The average ligand RMSD during the final 50 ns of both the original and replicate trajectories was calculated to evaluate convergence of the equilibrated binding mode. The combined ligand RMSD values were 3.89 ± 0.72 Å for IMPHY000687, 2.04 ± 0.77 Å for IMPHY007084, 5.53 ± 1.04 Å for IMPHY004619, 4.67 ± 1.15 Å for IMPHY012021, and 2.53 ± 0.34 Å for the reference inhibitor. Since the lower RMSD of the reference ligand was recorded in the final-50 ns calculation than the high RMSD noted in the first trajectory, this is attributed to the varying windows used in each trajectory. The high RMSD corresponds to the distance of the ligand from the initial docked position in the first trajectory, while the low RMSD in the final-50 ns trajectory corresponds to ligand movement in the late stage of the first and replicated trajectories. Therefore, the low RMSD does not invalidate the movement of the ligand from its initial position in the first trajectory.

The comparable ligand RMSD values obtained from the independent simulations indicate consistent ligand-binding behaviour and support the reproducibility of the molecular dynamics results despite the use of different initial velocity seeds.

#### 3.5.2. RMSF Analysis

##### Protein RMSF Analysis

**Figure 5:**
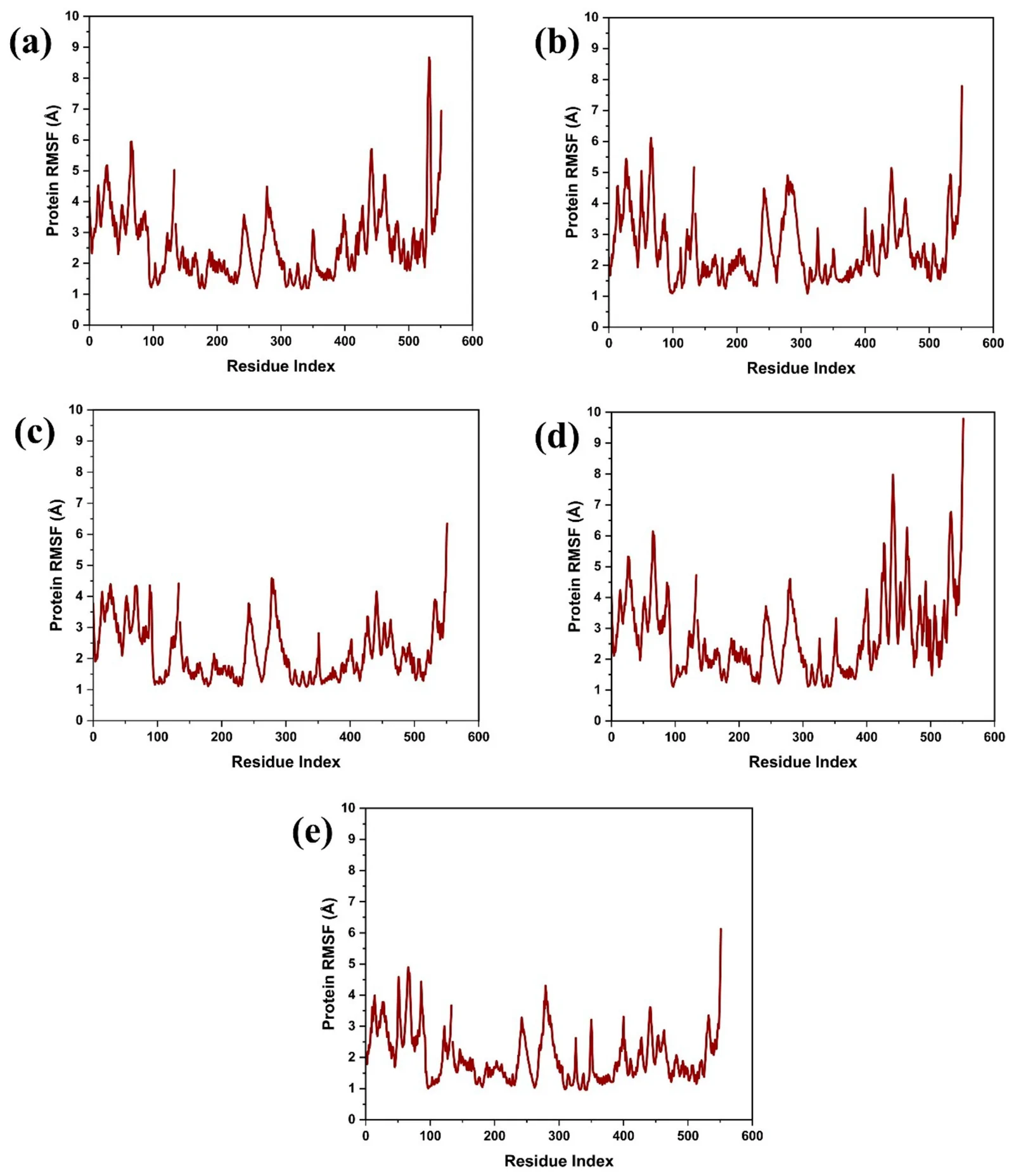
Protein RMSF of HIV-RT residues in complexes with (**a**) IMPHY000687, (**b**) IMPHY007084, (**c**) IMPHY004619, (**d**) IMPHY012021, and (**e**) reference inhibitor.

The protein RMSF analysis in all complexes showed that residues between 200 and 500 experienced major fluctuations with a value of 3 to 5 Å. Regions corresponding to the loop segments and terminal residues exhibited higher fluctuations, which is expected due to their intrinsic flexibility. In the case of the IMPHY004619 complex, the protein RMSF showed comparatively lower fluctuations in the core region, indicating limited internal fluctuations of the protein backbone. However, in the case of the protein in the IMPHY012021 complex, increased flexibility was observed in the 400 to 500 residue index regions, where the RMSF value reached 7–8 Å. Herein, core regions of the protein displayed relatively low fluctuations, suggesting that the overall structural integrity of the protein is maintained despite localized flexibility. The reference complex exhibited RMSF values were 3–4 Å, indicating limited internal fluctuations within the core region of the protein structure. Overall, the protein RMSF analysis indicates that ligand binding does not significantly alter the intrinsic flexibility pattern of the protein, as similar fluctuation regions are observed across all systems (Figure 5). The RMSF profile of the apo protein showed fluctuations in the same regions as in the complexed systems, with RMSF values ranging from 3–6 Å, indicating that these fluctuations are largely inherent to the protein structure. When interpreted alongside the RMSD results, the RMSF data suggest that the observed ligand-induced deviations arise primarily from rigid-body movement rather than significant internal structural deformation of the protein. (Supplementary Figure S3).

Protein RMSF analysis across all replica simulations demonstrated consistent fluctuation patterns with the primary trajectories. The major flexible regions, particularly within the 200–500 residue index range, were conserved across all complexes. The lower values of fluctuation (1–3 Å) were noted in the core parts, while the loops and terminal parts had higher fluctuation values (up to 7–8 Å). The location of flexible zones did not differ among all replicas, except that their magnitudes varied slightly. There were no abnormal deviations from the normal range in any trajectory of the replicas. These data indicate that the protein dynamics observed in the study are natural, and that different starting conditions do not influence them (Supplementary Figure S4).

##### Ligand RMSF Analysis

**Figure 6:**
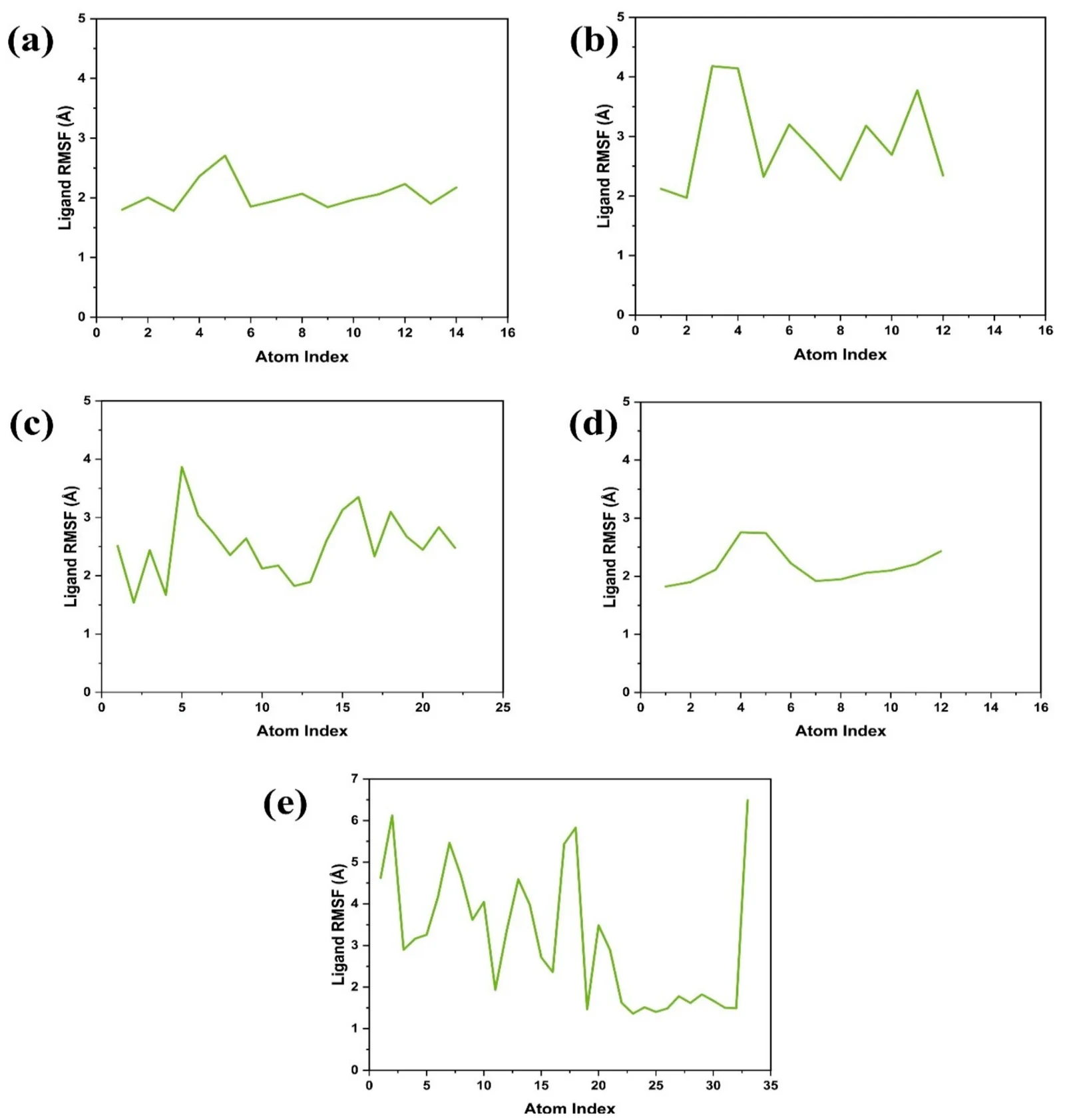
Ligand RMSF of compounds: (**a**) IMPHY000687, (**b**) IMPHY007084, (**c**) IMPHY004619, (**d**) IMPHY012021, and (**e**) reference inhibitor.

Ligand RMSF was used to evaluate the flexibility of the reference inhibitor and the bound phytochemicals throughout the simulation process. The IMPHY000687 ligand showed fluctuations in a range of 1.5–2.5 Å with minimal peaks; thus, the ligand showed low flexibility at the atom level. On the contrary, the IMPHY007084 ligand had higher RMSF values at particular atoms of the ligand, reaching 4–4.5 Å. The IMPHY004619 ligand RMSF showed several peaks in the range of 3–4 Å, indicating that there is moderate ligand flexibility at particular sites of the molecule. The IMPHY012021 ligand demonstrated relatively constrained fluctuations, with most atomic RMSF values less than 3.0 Å, indicating no significant deformations in the ligand structure. On the other hand, the reference inhibitor showed the highest RMSF values for some atoms, reaching more than 5.0 Å and demonstrating the flexibility of particular parts of the ligand. (Figure 6) In general, based on the RMSD and RMSF results, there were no significant conformational changes in the structures of the phytochemicals during the simulation. Thus, most deviations were connected with the rigid-body motions within the binding region.

The RMSF profiles for the ligand were further analyzed through independent replicates to test their reproducibility. Trends observed in the replicate trajectories were similar to those seen in the initial calculations, where the majority of atoms oscillated in the range of 1–3 Å, and larger oscillations (4–5 Å) were present in certain peripheral areas. Moreover, the spatial distribution of flexible atoms was nearly identical for both the replicate and primary simulations of all ligands.

In conjunction with the RMSD data, it can be concluded that the ligands maintain their structural integrity, and the deviations are mainly caused by rigid-body movements in the binding area rather than any substantial internal flexibility. Thus, the strong agreement between the initial and replicate simulations ensures the reproducibility of the ligand dynamics (Supplementary Figure S5).

### 3.6. Hydrogen Bond Analysis

**Figure 7:**
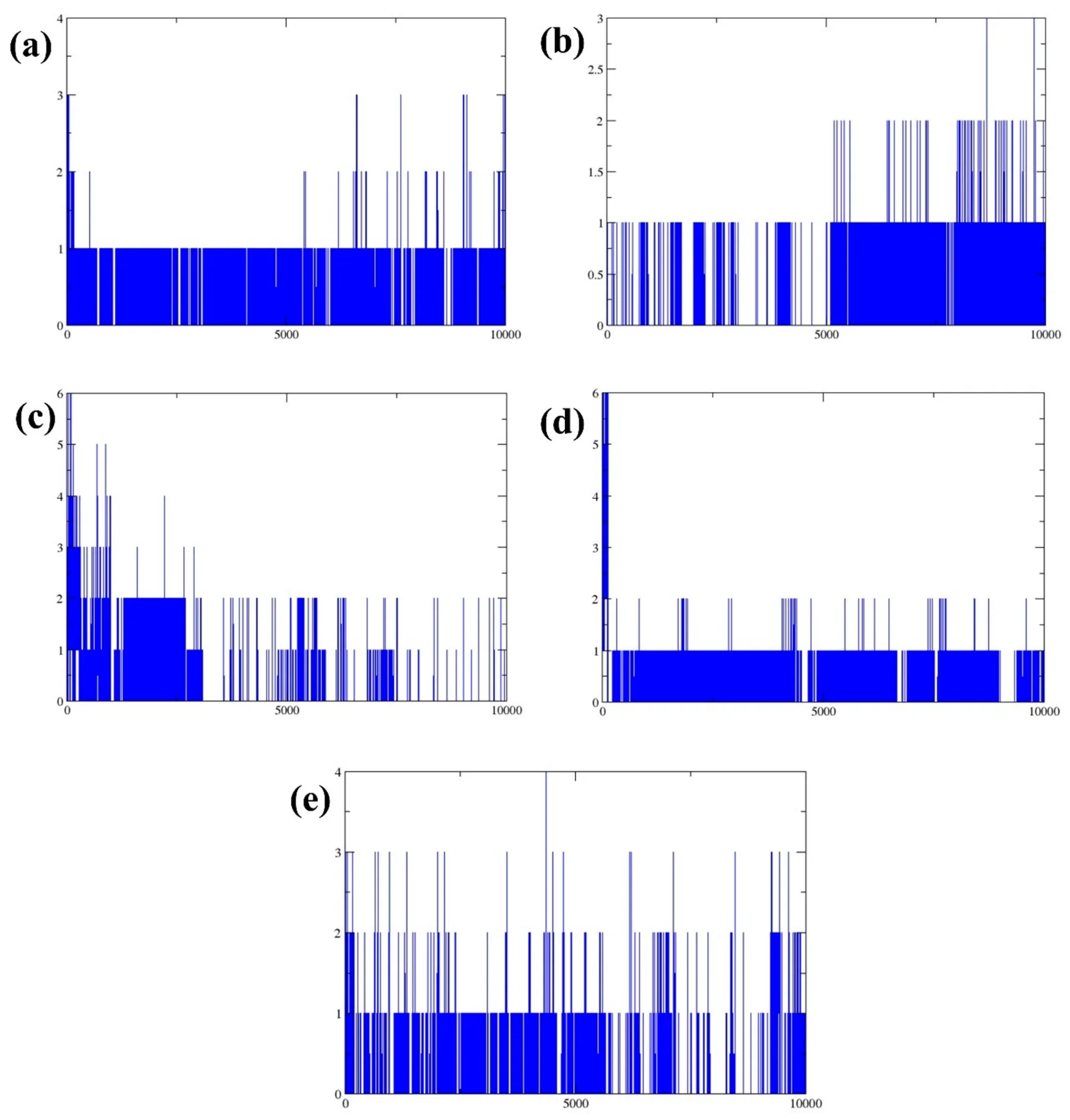
Time-dependent hydrogen-bond profiles of HIV-RT complexes with (**a**) IMPHY000687, (**b**) IMPHY007084, (**c**) IMPHY004619, (**d**) IMPHY012021, and (**e**) the reference inhibitor.

Interactions involving hydrogen bonding were analyzed to assess the presence and changes in intermolecular hydrogen bonds within each complex over time. In the case of IMPHY000687 complex, there is the formation of one hydrogen bond, which increases to two or three by the end of the simulation. Such dynamics suggest the presence of some temporal variations in interactions as a response to rearrangements of ligand positioning. In the IMPHY007084 complex, initially only one hydrogen bond is formed and increases to two in about fifty percent of the simulation, which shows the stabilization of interactions, followed by the repositioning of the ligand in the binding site. IMPHY004619 complex demonstrates a larger number of conformational changes compared to the other phytochemicals; at the beginning of the simulation, this complex has several hydrogen bonds formed (around 4–5). Thus, it may be concluded that many interactions occur initially, while the system stabilizes, with some interactions continuing during the simulation process. In the IMPH012021 complex, there is one hydrogen bond formed during the whole simulation process, with sporadic formation of others, showing the stable but dynamic protein–ligand interactions. However, in the case of the reference compound, there is an increase in fluctuation of hydrogen bonds from zero to three over the course of the simulation, which is associated with the lower consistency of interactions as compared to phytochemicals. Thus, based on the analysis of hydrogen bonds, it may be stated that phytochemicals tend to have consistent but dynamic interactions with the protein (Figure 7).

### 3.7. Last Pose Interaction Analysis

**Figure 8:**
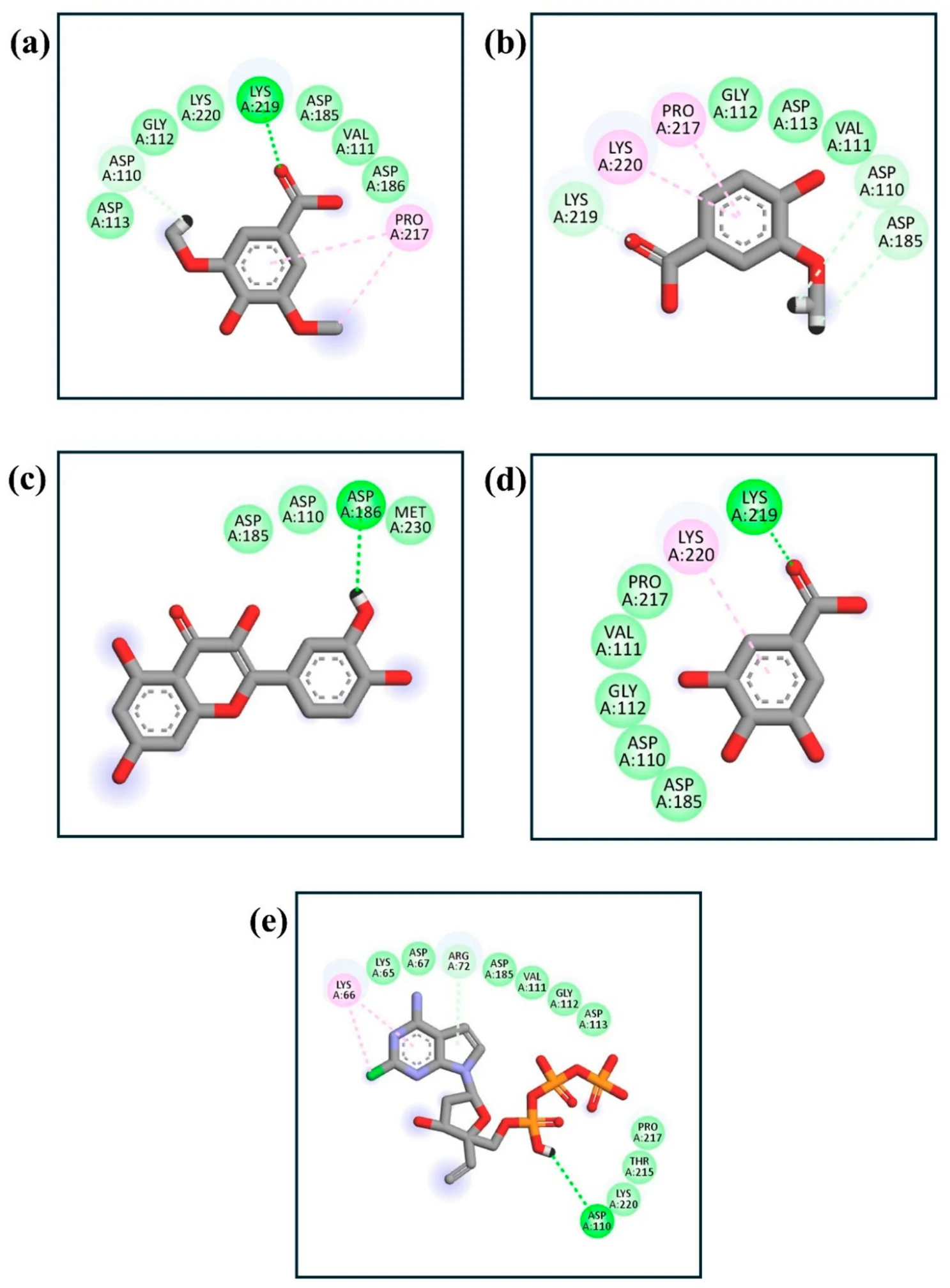
2D representation of the final pose obtained from molecular dynamics trajectories, showing interaction networks of HIV-RT complexes with (**a**) IMPHY000687, (**b**) IMPHY007084, (**c**) IMPHY004619, (**d**) IMPHY012021, and (**e**) reference inhibitor.

To analyze ligand–protein interactions at the end point of the molecular dynamics run, an interaction analysis was conducted for the last pose obtained from the trajectory. Thus, a hydrogen bond with Lys219 was noticed in the IMPHY000687 complex, while hydrophobic and π-associated interactions with Val111, Gly112, Asp113, Asp185, Asp186, and Lys220 were observed. No hydrogen bonds were observed in the last pose of the IMPHY007084 complex, although hydrophobic and π-associated interactions were present with Val111, Gly112, Asp113, Pro217, and Lys220. Similarly, the IMPHY004619 complex had a hydrogen bond with Asp186 and hydrophobic interactions with Asp110, Asp185, and Met230. Finally, for the IMPHY012021 complex, a hydrogen bond with Lys219 was detected together with hydrophobic interactions with Asp110, Val111, Gly112, Asp185, and Pro217, and a π-associated interaction with Lys220. The reference complex had a hydrogen bond with Asp110, as well as several hydrophobic and π-associated interactions with Lys65, Asp67, Val111, Gly112, Asp113, Asp185, Thr215, Pro217, and Lys220. It should be noted that these interactions relate to the last frame of the simulation and might not represent persistent interactions within the trajectory. This needs to be taken into account, considering the results of the RMSD analysis, which reveals considerable ligand mobility in some complexes. Therefore, the last-pose interactions can be considered temporary conformations rather than evidence of stable binding. Overall, the analysis of docking-derived and last MD pose interactions indicates a change in the pattern of interactions and repositioning of the ligands in the binding area during the simulation (Figure 8 and Table 3).

### 3.8. Principal Component Analysis

**Figure 9:**
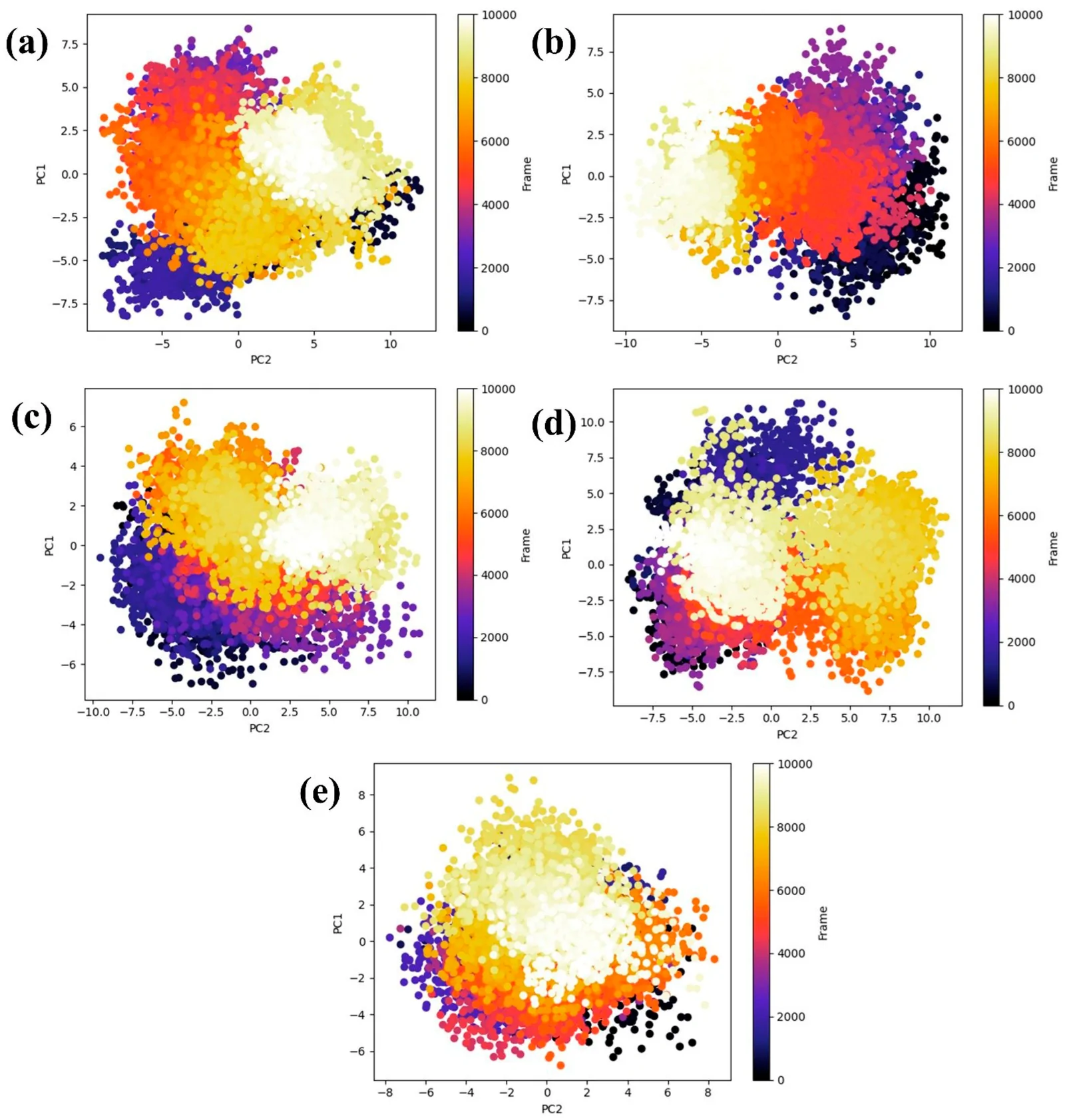
Projection of molecular dynamics trajectories onto the first two principal components (PC1 and PC2) for HIV-RT complexes with (**a**) IMPHY000687, (**b**) IMPHY007084, (**c**) IMPHY004619, (**d**) IMPHY012021, and (**e**) reference inhibitor.

PCA analysis was used to detect the major motions of HIV-RT protein with Oregano phytochemicals, the reference inhibitor, and in the apo form. The first two principal components (PCs) characterize the main modes of motion. The conformer distribution of the IMPHY000687 complex was relatively compact with smooth transitions in both PC1 and PC2 directions. The lack of separation in the distribution demonstrates a smooth transition of the conformation rather than any structural reorganization. In the IMPHY007084 complex, a relatively broad distribution was observed, especially in the PC2 direction, which suggests high conformational sampling and more heterogeneity. The profile of the IMPHY004619 complex was characterized by a clustering of trajectory frames, meaning relatively limited conformational changes. In contrast, the IMPHY012021 complex had several overlapping conformational clusters with smooth transitions, indicating moderate conformational flexibility and the ability to access several stable conformations within one phase space region. The reference inhibitor complex had a more spread distribution in the PC1-PC2 space, with distinct regions corresponding to particular stages of simulations and more conformational heterogeneity (Figure 9). The apo protein (Supplementary Figure S6) occupied the largest area in the PC1-PC2 space, suggesting maximal conformational sampling in the absence of any ligand.

### 3.9. Free Energy Landscape (FEL) Analysis

**Figure 10:**
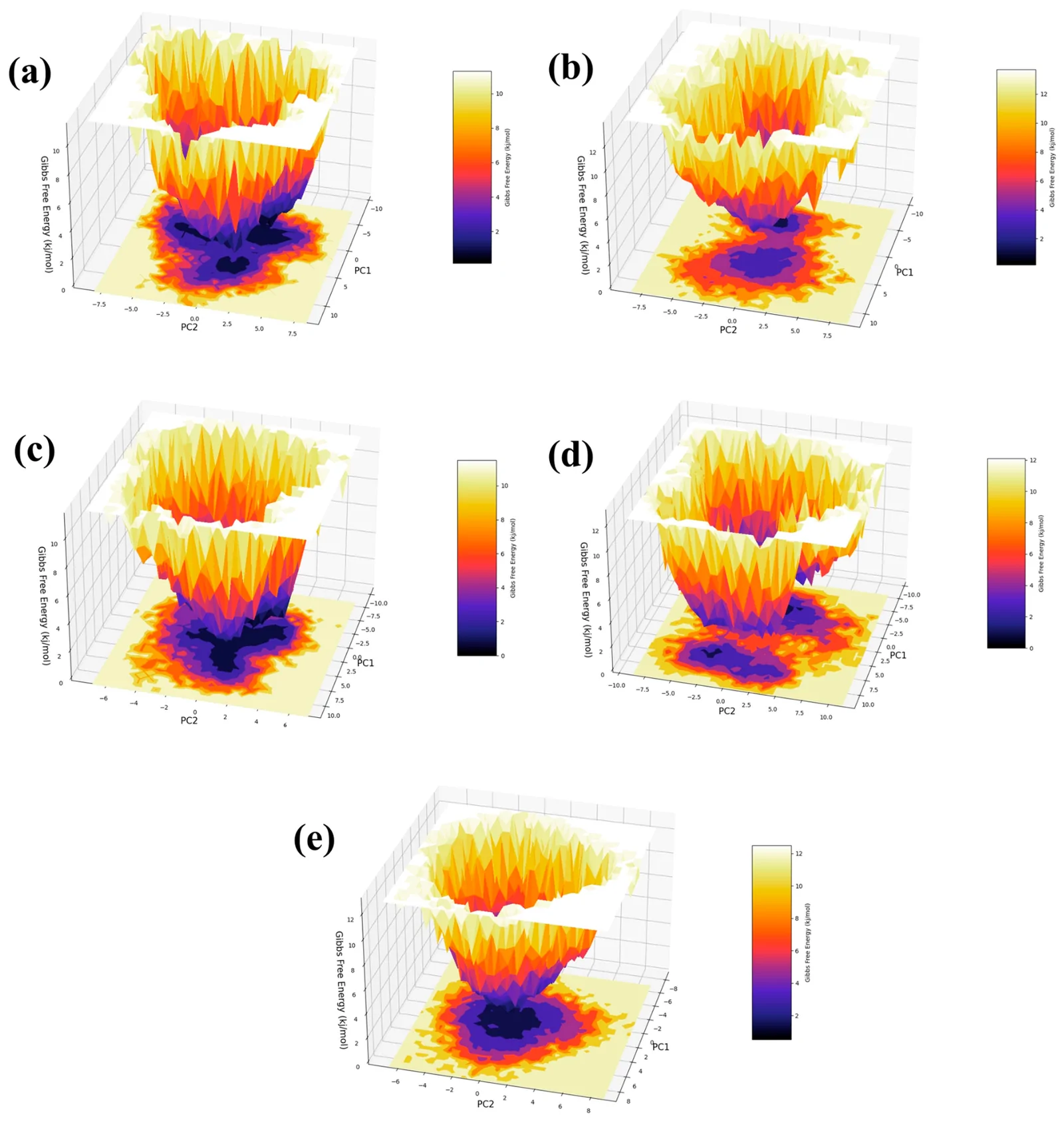
Projection of FEL along PC1 and PC2 for HIV-RT complexes: (**a**) IMPHY000687; (**b**) IMPHY007084; (**c**) IMPHY004619; (**d**) IMPHY012021; (**e**) reference inhibitor.

The conformational preferences and thermodynamic stability of HIV-RT in complex with the selected apo protein, phytochemicals, and reference inhibitor were investigated using FEL analysis. PC1 and PC2 generated FELs, and the 3D FEL plot is shown in Figure 10. In all the plots, the Gibbs free energy values (kJ/mol) are represented by colour scales where the dark blue regions represent low energy (0 kJ/mol) or thermodynamically favourable conformation, and the higher energy (4–12 kJ/mol) (less stable states) is represented by green, white, and yellow.

Conformational sampling becomes increasingly limited as the basin becomes narrow and localized. On the other hand, wider or multibasin landscapes are an indication of greater conformational diversity and transitions between metastable states. IMPHY000687 complex has a well-defined energy basin located in the narrow space of PC1-PC2, indicating limited conformational sampling and few large-scale transitions. In turn, the IMPHY007084 complex has a number of low-energy basins separated from each other by shallow energy barriers, which is evidence of the presence of several available conformational states. In the same way, the complex of IMPHY004619 shows a well-defined energy minimum with low dispersion and restricted conformational sampling in the localized region of the phase space. For IMPHY012021, the energy landscape is characterized by a moderately well-defined global minimum and adjacent low-energy basins. In this case, the energy landscape indicates a regulated conformational transition between the energetically close states. Comparing the inhibitor, one can see that its energy landscape is characterized by the existence of a great number of shallow minima in different locations on the energy landscape. This indicates increased conformational diversity and wider conformational sampling of the complex. The apo protein energy landscape is the most dispersed and widely distributed energy distribution, with the presence of many shallow basins in the space of PC1 and PC2. FEL analysis characterizes the conformational energy landscape of the protein but does not provide any data on ligand-binding stability. Energy basins correspond to preferential and transitional conformations of the protein under various simulation conditions (Supplementary Figure S7).

**Figure 11:**
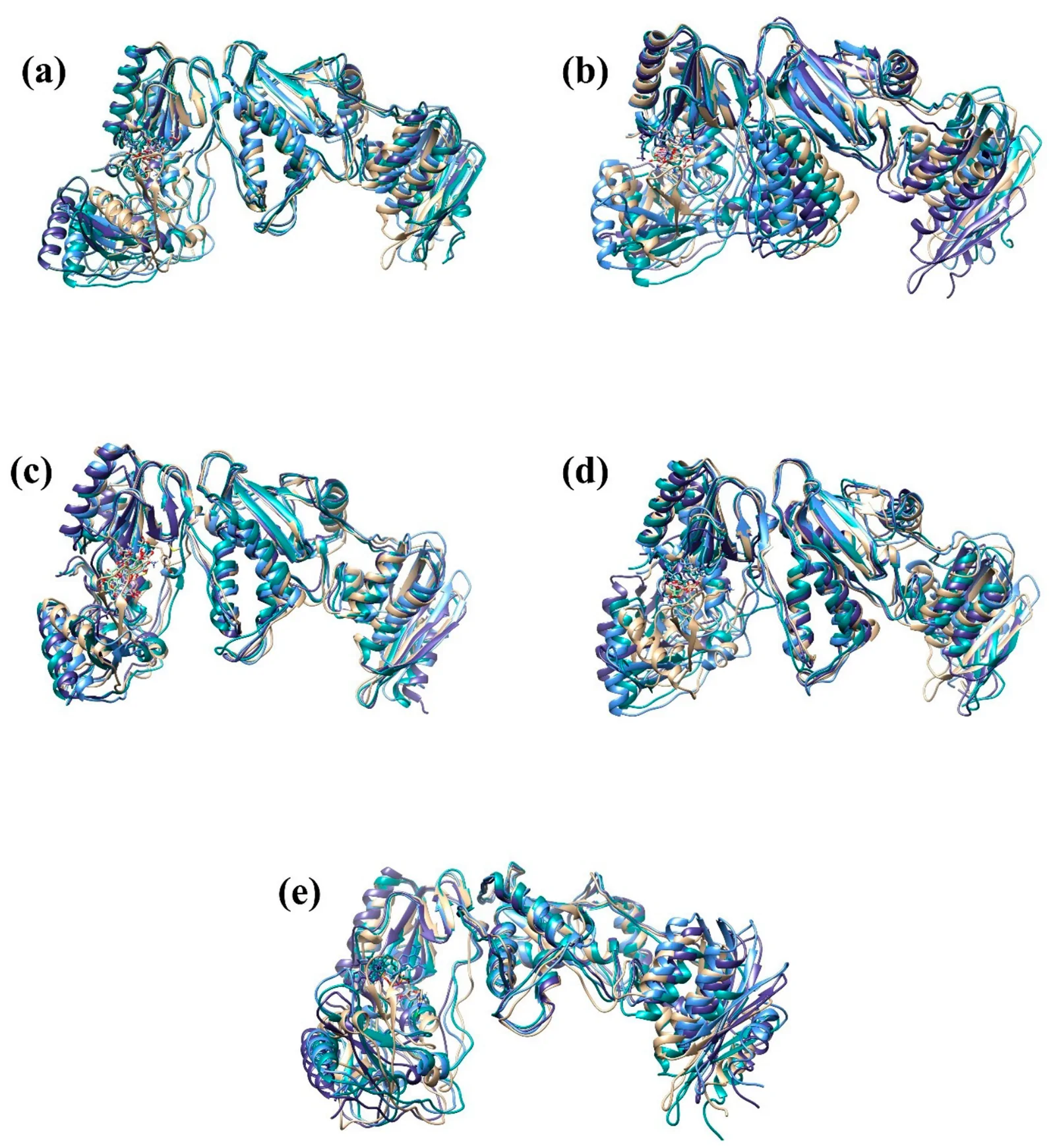
Superimposed global minimum binding conformations of HIV-RT complexes obtained using FEL: (**a**) IMPHY000687, (**b**) IMPHY007084, (**c**) IMPHY004619, (**d**) IMPHY012021, and (**e**) reference inhibitor.

The structures representing global minimum energy basins were isolated and aligned structurally to assess structural similarity within the lowest-energy conformations sampled in FEL calculations. The RMSD values obtained for the lowest energy basins reflect structural similarity within the dominant conformational states simulated. For the IMPHY000687 complex, the RMSD for superposed global minimum structures was 2.070 Å, indicating high structural similarity within the dominant low-energy basin. The complex of IMPHY007084 showed a somewhat higher RMSD value of 2.231 Å, which corresponds to a wider distribution in FEL and moderate conformational diversity in the low-energy region. The RMSD value for the IMPHY004619 phytochemicals was 2.124 Å, suggesting that the conformational sampling of the molecule occurs in a restricted region of phase space. The superposition of the global minimum structures resulted in a 2.295 Å RMSD for IMPHY012021, showing somewhat increased conformational dispersion but still staying within the same ensemble. The superposition of global minimum structures resulted in an RMSD of 2.178 Å for the reference inhibitor, indicating similar structural similarity in low-energy conformations. The difference in behavior was caused by conformational sampling within the energy landscape and not by ligand stability in binding sites. Overall, the results suggest that the protein adopts similar structural conformations within low-energy basins, and the conformational dispersion level differs for particular systems (Figure 11 and Figure S8).

### 3.10. QM/MM Analysis

**Figure 12:**
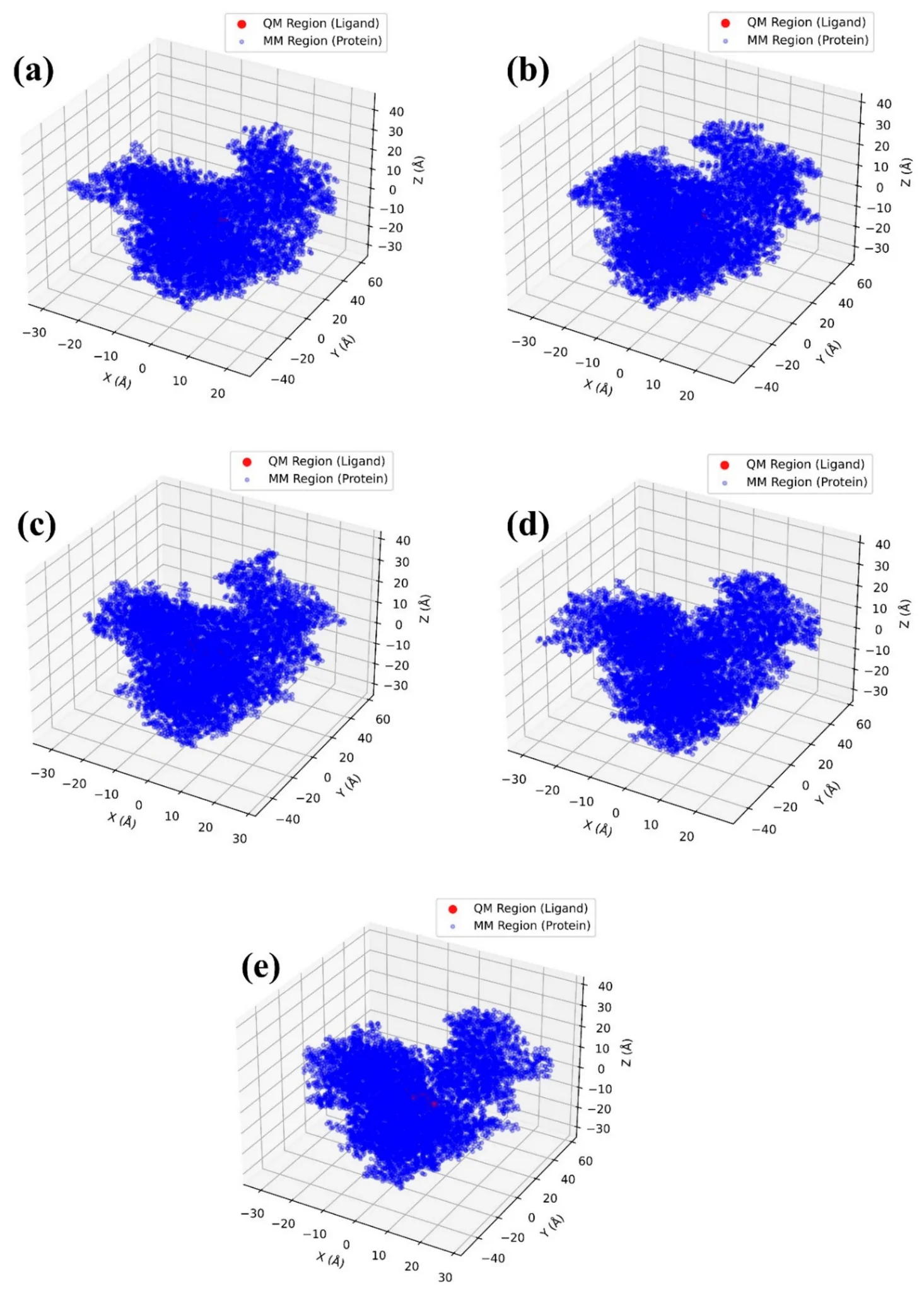
QM/MM analysis of the electronic characteristics of HIV-RT complexes with (**a**) IMPHY000687, (**b**) IMPHY007084, (**c**) IMPHY004619, (**d**) IMPHY012021, and (**e**) reference inhibitor.

To gain more information about the electronic properties of protein–ligand complexes in HIV-RT, quantum mechanics/molecular mechanics (QM/MM) analysis was conducted. In this framework, the ligand is described at the quantum mechanical level, while the protein environment surrounding the ligand is characterized at the molecular mechanics level. Thus, the possibility of investigating the electronic properties of the system is provided. Figure 10 depicts the distribution of the QM and MM areas for all complexes. For example, the IMPHY000687 complex has a QM/MM total energy value of approximately −1.97 × 10^4^ eV. Analogously, the QM/MM total energy value for the IMPHY007084 complex is about −1.66 × 10^4^ eV, whereas those for the IMPHY004619 and IMPHY012021 complexes are equal to −3.01 × 10^4^ eV and −1.76 × 10^4^ eV, respectively. The absolute QM/MM total energy of the reference inhibitor was found to be around −8.48 × 10^4^ eV. Absolute QM/MM total energies, however, are very much dependent on the chemical composition, size of the system, and number of electrons; thus, they cannot be used for comparing the affinity of the ligands under investigation. It is worth mentioning that QM/MM total energies are extremely sensitive to system size and number of electrons; therefore, absolute energy values cannot be used as criteria for binding or interaction strength among the studied ligands. In consequence, the QM/MM calculations were analyzed as qualitative data regarding the electronic properties of the individual complexes, and not as quantitative comparisons of the affinity for binding of the ligands. The differences in total energies mainly depend on the composition and size of the system, but not on the binding strength (Figure 12).

### 3.11. Machine Learning-Based Prediction

**Figure 13:**
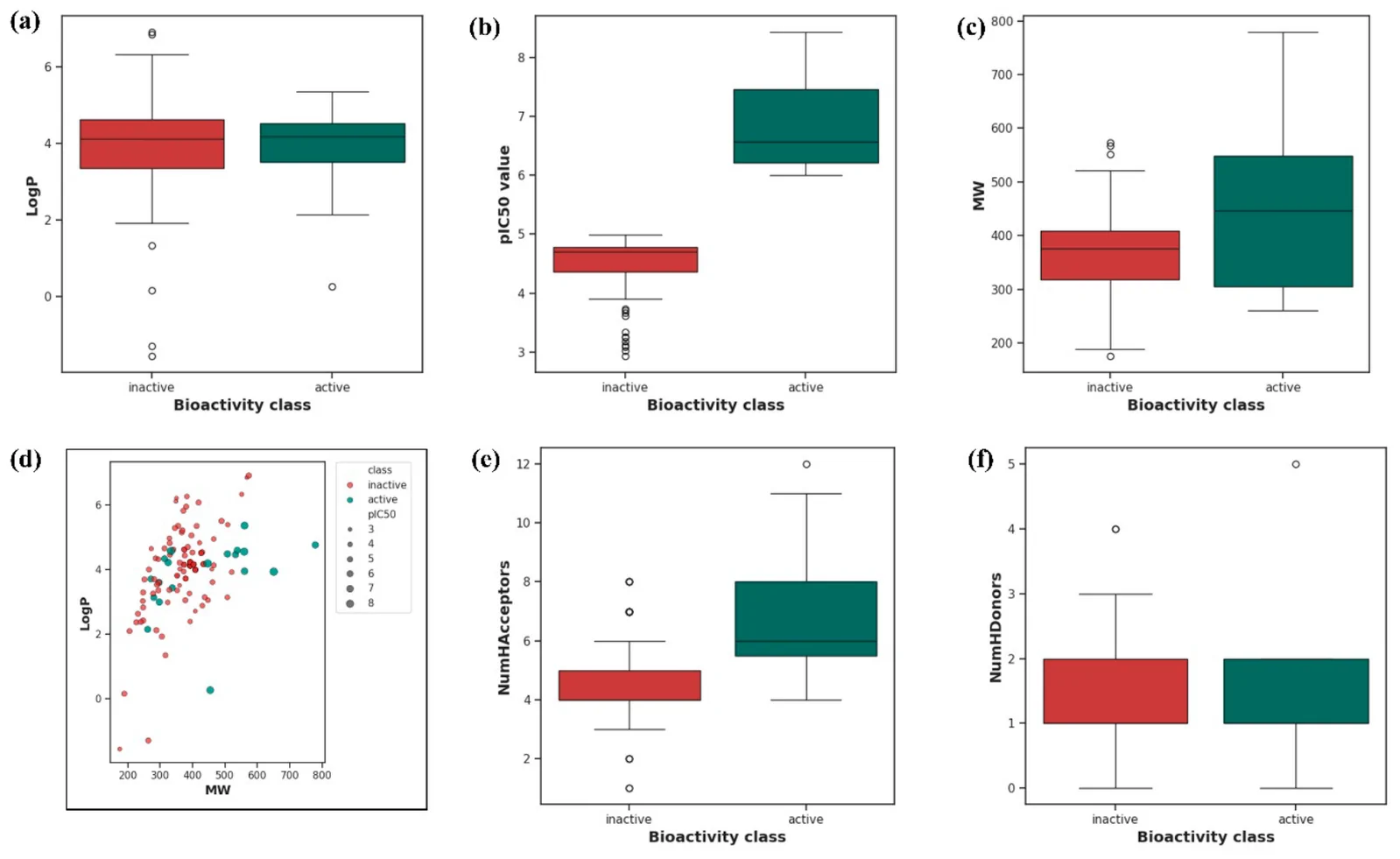
Distribution of key molecular descriptors for 165 HIV-RT inhibitors. (**a**–**c**) Boxplots of LogP, pIC_50_, and molecular weight (MW) by activity class. (**d**) Scatterplot of LogP vs. MW, sized by pIC_50_ and colored by activity. (**e**,**f**) Boxplots of hydrogen bond acceptors (HBA) and donors (HBD). Actives show higher pIC_50_, MW, and HBA counts, suggesting key features linked to RT inhibition.

For feature analysis, a set of 165 HIV-1 reverse transcriptase inhibitors, which were validated by experimental results, was collected from the ChEMBL database. The dataset was divided into active and inactive compound groups based on the pIC_50_ ≥ 6.0 criterion. The comparison of important molecular descriptors showed that active and inactive HIV-1 reverse transcriptase inhibitors have different physicochemical properties. As shown in Figure 13a–c, active HIV-1 reverse transcriptase inhibitors had higher lipophilicities, higher molecular weights, and higher pIC_50_ values compared to inactive HIV-1 reverse transcriptase inhibitors. Active HIV-1 reverse transcriptase inhibitors had average molecular weights of 500 Da, while inactive HIV-1 reverse transcriptase inhibitors had average molecular weights in the range of 350–400 Da. The scatter plot of the relationship between the logarithm of partition coefficient (LogP) and molecular weight, which was divided by pIC_50_, showed that active HIV-1 reverse transcriptase inhibitors had higher potency and favourable physicochemical properties, with LogP values ranging from 3 to 5 and molecular weights ranging from 400 to 600 Da, as shown in Figure 13d. Hydrogen bond acceptors and hydrogen bond donors were used to distinguish active and inactive HIV-1 reverse transcriptase inhibitors. Active HIV-1 reverse transcriptase inhibitors had higher values of hydrogen bond acceptors, which were in the range of 6 to 10, compared to inactive HIV-1 reverse transcriptase inhibitors, as shown in Figure 13e. Hydrogen bond donors had slightly higher values in active HIV-1 reverse transcriptase inhibitors compared to inactive HIV-1 reverse transcriptase inhibitors, as shown in Figure 13f.

### 3.12. Model Evaluation and Prediction Performance

**Figure 14:**
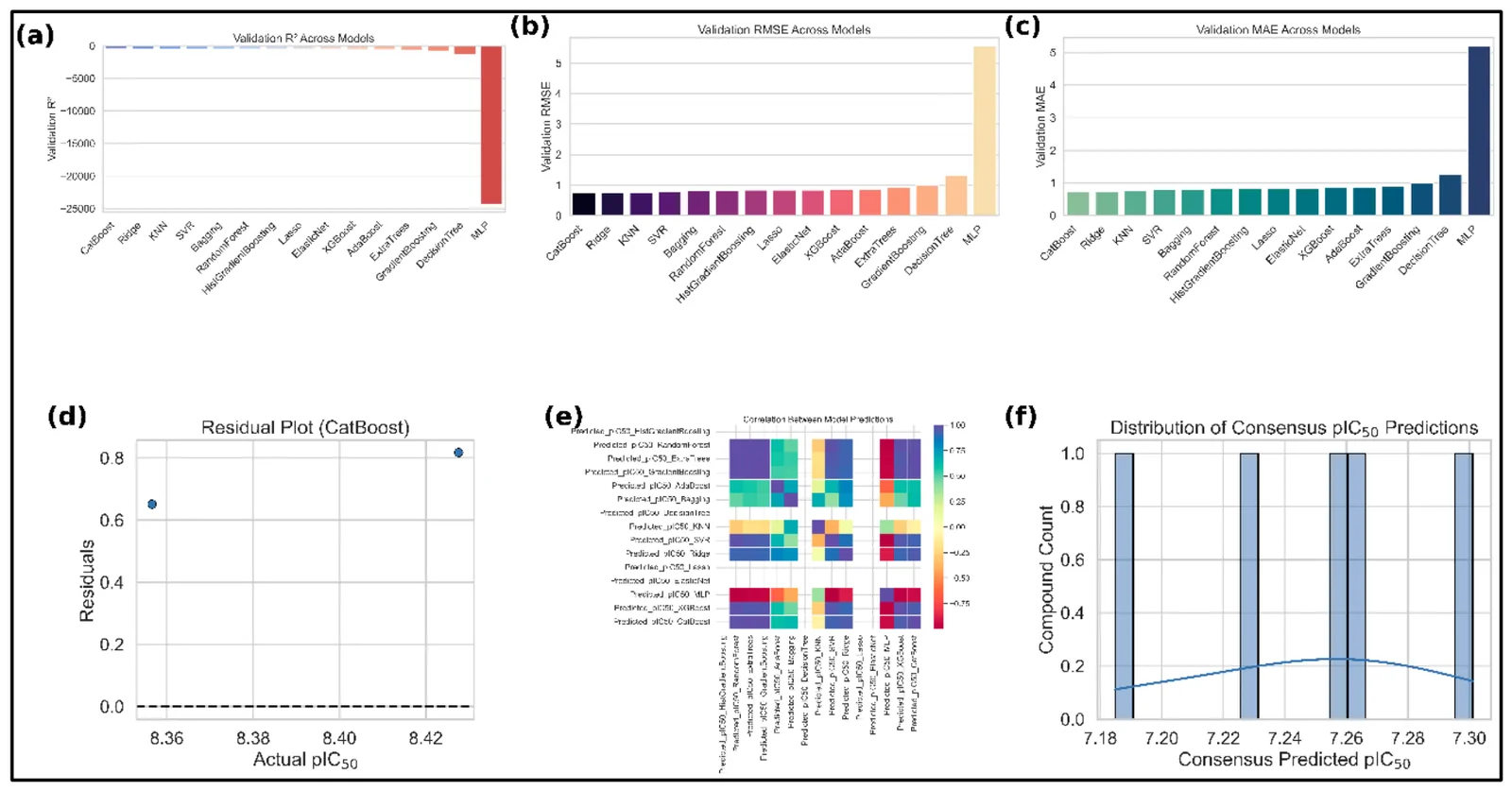
Machine learning model evaluation and activity prediction. (**a**) Validation R^2^, (**b**) validation RMSE, (**c**) validation MAE of the evaluated regression models, (**d**) residual plot of the CatBoost model, (**e**) correlation heatmap of predicted pIC_50_ values across the evaluated models, and (**f**) distribution of CatBoost-predicted pIC_50_ values for the shortlisted phytochemicals and the reference inhibitor.

The predictive ability of the developed QSAR models was tested via an 80:20 train/test ratio in combination with a five-fold cross-validation procedure. CatBoost from the set of selected regression models was chosen for the prediction of shortlisted phytochemicals due to low validation error rates. The RMSE and MAE values obtained during the validation step were 0.739 and 0.734 correspondingly. Additionally, the five-fold cross-validation was conducted to evaluate model performance variability for various subsets of training data. Given the rather small size of the experimental data, the analysis results were taken into account with caution, and the QSAR predictions served as one part of the whole computational analysis process. The reference inhibitor predicted the maximum inhibitory activity with pIC_50_ value of 7.49‚ followed by IMPHY004619 (7.40)‚ IMPHY007084 (7.37)‚ IMPHY012021 (7.36)‚ and IMPHY000687 (7.35). The values of the projected pIC_50_ of phytochemicals shortlisted are close to each other (7.35–7.49)‚ indicating that these phytochemicals have similar predicted inhibitory activity. This further shows the usefulness of prioritizing them for structural analysis. Figure 14d summarizes the distribution of prediction errors (residuals) across all tested regression algorithms applied to the CatBoost model‚ indicating that most residuals fell in the range of ±0.8 pIC_50_ units without any apparent systematic bias. Figure 14e shows the correlation matrix of the predicted pIC_50_ values obtained from the different regression algorithms for the shortlisted compounds. The distribution of predicted pIC_50_ values shows that all selected compounds were clustered within a similar activity range (Figure 14f).

For the evaluation of the robustness of the prediction, applicability domain (AD) was estimated with leverage- and distance-based approaches. IMPHY007084, IMPHY012021, and the reference inhibitor are found within the AD, which indicates the reliability of their predictions in the chemical space covered by the training set. IMPHY000687 and IMPHY004619 were identified to be outside the AD, suggesting that the predicted pIC_50_ values are based on the extrapolations to less-covered parts of the chemical space and should be interpreted with caution. It is worth noting that the choice of these molecules was not based only on the machine learning approach, but also on the whole computational workflow with molecular docking, MD simulations, QM/MM, DFT, and ADMET calculations. Therefore, the AD estimation adds one more confidence factor in the interpretation of QSAR predictions and provides evidence for the appropriate use of machine learning as a complementary tool in the prioritization process (Supplementary Table S2 and Figure S10).

## 4. Discussion

This research work aimed to identify the phytochemical inhibitors obtained from oregano against HIV-RT using computational approaches. The reference ligand used in the research MK 8527 is the recently identified nucleoside RT translocation inhibitor with an IC_50_ value of 0.21 nM [9]. From the 820 phytochemical compounds, the top four candidates IMPHY000687, IMPHY007084, IMPHY004619, IMPHY012021) displayed docking energy around −9.23 to −9.42 Kcal/mol and re-docking around −9.18 to −9.34 Kcal/mol. In a recent study, the docking score of −9.8 to −12.7 kcal/mol for the imidazopyridine–Schiff base synthesized compounds, compared with controls nevirapine (−9.5 Kcal) and didanosine (−8.0 Kcal) [45].

The DFT analysis showed that the selected phytochemicals possess comparable electronic characteristics, with HOMO–LUMO energy gaps ranging from 4.03 to 4.73 eV, while the reference inhibitor exhibited a slightly higher gap (4.89 eV). Among the phytochemicals, IMPHY004619 displayed the lowest gap, indicating relatively higher electronic reactivity; however, the overall variation across compounds is small. Smaller HOMO-LUMO energy gaps are generally related to higher chemical reactivity, but it does not directly imply better inhibitory ability. Similar energy gap values mean that the molecules have comparable electronic properties. On the other hand, binding efficiency is determined by parameters besides electronic structure, including structural complementarity and dynamics in the protein medium. Furthermore, since DFT calculations were conducted without dispersion correction, interactions like π-π stacking and hydrophobic interactions are qualitative and do not appear in the DFT results. Overall, DFT data better represent electronic properties than binding efficiency. The interaction analysis showed major hydrophobic interactions and hydrogen bonds. The residues Val111, Gly112, Asp110, Asp113, and Pro217 participated in major interaction. The presence of hydrophobic interactions has been reported in the previously identified inhibitors [46]. However, ligand-induced changes were observed from the MD simulations, instead of uniformly high stability in all complex systems studied. While a few phytochemicals displayed relatively low deviations in certain trajectory segments compared to their docked structures, many experienced notable shifts in position as compared to their original docked structure. This was also observed in the reference ligand MK-8527, which demonstrated considerable movement with a ligand RMSD of about 10–14 Å in the main trajectory. The RMSF profiles further indicated that the major fluctuations were localized to solvent-exposed loop regions, whereas residues surrounding the inhibitor-binding pocket remained comparatively stable, suggesting that ligand binding did not induce substantial structural perturbations. To further evaluate the reproducibility of the simulations, independent replicate molecular dynamics simulations were performed using different initial velocity distributions. The comparable ligand RMSD values obtained during the final 50 ns of the original and replicate trajectories (3.89 ± 0.72 Å for IMPHY000687, 2.04 ± 0.77 Å for IMPHY007084, 5.53 ± 1.04 Å for IMPHY004619, 4.67 ± 1.15 Å for IMPHY012021, and 2.53 ± 0.34 Å for the reference inhibitor) demonstrate consistent dynamic behaviour across independent simulations.

The selected phytochemicals, especially IMPHY007084 showed adaptive conformational shift with higher protein RMSD at the second phase of simulation, followed by final stabilisation. This indicates the positional rearrangement of the ligand within the binding site rather than complex destabilisation. This kind of adoptive conformational behaviour has been previously identified in NNRTIs, where the loop and domain region contribute to induce fit mechanism [47]. It has been proven that stable binding in the protein–ligand complex still exists even with structural fluctuation within the binding site. The PCA and FEL results further confirmed these findings by exhibiting the ligand conformational shift. In the FEL analysis, the complex and apo showed dispersed energy patterns, and this clearly points to the stabilising effect of each ligand. In this investigation, the phytochemical compounds tend to restrict conformational motion and support the energetically stable pose. Moreover, the low RMSD values obtained after the superimposition of global minima confirmed the convergence along with stable conformations.

The QM/MM calculations provided additional insight at the electronic level on the specific protein–ligand complexes compared to that acquired through the classical MD simulations. In view of the fact that the total energies calculated were dependent on the composition of the molecule, size of the system, and number of electrons, these were evaluated qualitatively and not quantitatively. Lastly, machine learning-based QSAR prediction was performed to minimise the false values. Using the pre-built set of HIV-RT inhibitors, the prediction of pIC_50_ values for modern RT is close to the selected compounds. Overall, when the reference ligand is compared against the identified inhibitor reports sub-nanomolar translocation inhibitors (MK-8527) and nanomolar NNRTIs (DAPY and pyrimidine derivatives, and BPPT); together, these computational results justify the importance of selecting these phytochemicals as potential HIV-RT inhibitory molecules to be further validated experimentally.

The findings of the present study are in strong agreement with recent advances in computational approaches for targeting HIV-1 reverse transcriptase inhibitors. Previous studies have demonstrated that tripeptide-based inhibitors, such as FHW, exhibit significantly high binding affinity, even surpassing standard drugs like Nevirapine, highlighting the potential of peptide-based therapeutic strategies [10]. Through concurrent phytochemical screening, several molecules from plants, such as rosmarinic acid, arctigenin, luteolin, and quercetin, have been found to be potential inhibitors of HIV-RT. In addition, large-scale computational screening of more than 85 phytochemicals with targets such as HIV-RT [48] further supports the reliability of computational drug discovery. With recent artificial intelligence tools employed in drug discovery pipelines, several molecules have been screened from millions of molecules and identified as novel NNRTIs that inhibit wild-type as well as mutant forms of the enzyme [49]. In addition, studies carried out on plants have identified anti-HIV-1 RT activity among various phytochemicals isolated from more than 132 plants. Collectively, these studies corroborate the present findings and emphasize that integrating artificial intelligence, QSAR modelling, and molecular simulation techniques with phytochemical libraries provides a robust and efficient strategy for discovering novel HIV-RT inhibitors [48].

Despite the advanced computational framework, this research has certain limitations. The first one is that the conclusion is mainly based on in silico analysis, with no experimental validation through enzyme inhibition assays and cell-culture studies. In addition, clinically relevant resistance-associated HIV-RT mutants were not investigated; therefore, the activity of the selected phytochemicals against drug-resistant HIV cannot be inferred from the present computational findings. Moreover, in the present study is that docking and molecular dynamics simulations were performed using the isolated p66 catalytic subunit. Consequently, the complete p66/p51 heterodimer, nucleic acid, and metal-ion environment associated with the HIV-RT translocation mechanism was not explicitly modelled. Thus, the current results can only be viewed as the computational identification of putative ligand interaction with the MK-8527 binding site of the p66 subunit and not as the inhibition of or mechanism for HIV-RT translocation. Next, the MD simulations were performed against a single HIV-RT and did not account for clinically relevant mutations. Although in silico ADMET analysis was performed to predict the pharmacokinetic and toxicity profiles of the selected phytochemicals, these predictions were not experimentally validated. Therefore, pharmacokinetic properties, including bioavailability, metabolic stability, toxicity, and efficacy, should be confirmed through in vitro and in vivo studies before considering clinical applications.

## 5. Conclusions

In this study, a computational pipeline was applied to identify potential HIV-RT inhibitory candidates from *Origanum vulgare* phytochemicals. The selected compounds had unique dynamics of interactions within the HIV-RT binding site studied, with different levels of structural and positional reorganization in MD simulation. Moreover, the predicted inhibitory activity compared to the reference compound indicates that these phytochemicals (IMPHY000687, IMPHY007084, IMPHY004619, and IMPHY012021) are potential HIV-RT inhibitory candidates. These results were supplemented by QM/MM computations, which gave electronic-level information about the protein–ligand systems, as well as by QSAR predictions for the selected molecules using machine learning. This investigation provides computational support for the potential inhibitory activity of the selected compounds; however, future studies should focus on experimental validation to strengthen the current findings. Expansion of the machine learning models with diverse HIV-RT inhibitors will also enhance the reliability and support the structural optimization of these compounds towards significant therapeutic development. Overall, the integrated computational findings prioritize the selected phytochemicals as potential HIV-RT inhibitory candidates for further experimental investigation. Their inhibitory activity requires experimental validation, and their effectiveness against drug-resistant HIV cannot be established from the present study because resistance-associated RT mutants were not evaluated.

## Figures and Tables

**Table 1:** ADME/T Characteristics of Top-Ranked Compounds.

Compound	Lipinski Rule	HIA (Probability)	BBB Permeability	NP-Likeness Score	Volume Distribution (VD)	CYP3A4 Inhibitor	AMES Toxicity
IMPHY000687	Accepted	0.028	0.457	0.544	0.459	0.0032	0.009
IMPHY007084	Accepted	0.013	0.439	0.524	0.358	0.042	0.015
IMPHY004619	Accepted	0.014	0.008	1.701	0.579	0.943	0.657
IMPHY012021	Accepted	0.085	0.099	0.981	0.466	0.023	0.053

**Table 2:** Intermolecular interactions of selected docked complexes.

S No.	Complex	H-Bond	H-Bond Length	Hydrophobic	π-π Stacking/ π-π Cation/π-Alkyl
1	IMPHY000687	Lys^66^, Asp^67^,	Lys^66^(4.47 Å) Asp^67^(4.26 Å)	Val^111^, Gly^112^, Ala^114^,	Lys^65^, Lys^70^, Arg^72^, Asp^110^, Lys^220^,
2	IMPHY007084	Lys^66^, Asp^67^,	Lys^66^(4.47 Å) Asp^67^(4.28 Å)	Lys^70^, Val^111^, Gly^112^, Ala^114^,	Lys^65^, Arg^72^, Asp^110^, Lys^220^,
3	IMPHY004619	Ala^114^, Asp^185^, (Asp^186^)^2^,	Ala^114^(3.98 Å) Asp^185^ (4.17 Å) Asp^186^ (3.82 Å) Asp^186^ (3.58 Å)	Asp^110^, Val^111^, Asp^113^, Tyr^115^, Phe^116^, Gln^151^, Tyr^183^, Met^184^, Met^230^,	(Arg^72^)^2^, (Asp^185^)^2^,
4	IMPHY012021	Lys^66^, Asp^67^,	Lys^66^(4.31 Å) Asp^67^(4.26 Å)	Lys^70^, Val^111^, Gly^112^, Ala^114^, Asp^185^,	Lys^65^, Arg^72^, Asp^110^, Lys^220^,
5	Control	(Lys^65^)^2^, (Arg^72^)^2^, (Asp^110^)^2^, Ala^114^,	Lys^65^ (4.49 Å) Ala^114^(4.22 Å)	Ile^63^, Leu^74^, Val^111^, Gly^112^, Asp^113^, Gln^151^, Gly^152^, Lys^220^,	Arg^72^, (Ala^114^)^2^, (Tyr^115^)^2^, (Met^184^)^2^,

**Table 3:** The last pose interaction of the simulated complexes.

S No.	Complex	H-Bond	Hydrophobic	π-π Stacking/ π-π Cation/π-Alkyl
**1**	IMPHY000687	Lys^219^,	Val^111^, Gly^112^, Asp^113^, Asp^185^, Asp^186^, Lys^220^,	(Pro^217^)^2^
**2**	IMPHY007084	--	Val^111^, Gly^112^, Asp^113^,	Pro^217^, Lys^220^,
**3**	IMPHY004619	Asp^186^,	Asp^110^, Asp^185^, Met^230^,	--
**4**	IMPHY012021	Lys^219^,	Asp^110^, Val^111^, Gly^112^, Asp^185^, Pro^217^	Lys^220^,
**5**	Control	Asp^110^,	Lys^65^, Asp^67^, Val^111^, Gly^112^, Asp^113^, Asp^185^, Thr^215^, Pro^217^, Lys^220^,	(Lys^66^)^2^,

## Data Availability

The datasets used and/or analyzed in the current study are available in public repositories, such as the Protein Data Bank (https://www.rcsb.org/structure/9DM9, accessed on 5 December 2025) and the IMPPAT Database (https://cb.imsc.res.in/imppat/home, accessed on 5 December 2025). The corresponding IDs or accession numbers are provided in the main manuscript and the Supplementary Materials; further inquiries can be directed to the corresponding authors.

## Supplementary Materials

The following supporting information can be downloaded at: https://www.mdpi.com/article/10.3390/v18091010/s1, Table S1: List of compounds along with their docking scores after virtual screening; Table S2: Leverage- and distance-based applicability domain (AD) analysis indicating the prediction reliability of the shortlisted phytochemicals and the reference inhibitor; Figure S1: RMSD of apo-protein; Figure S2: RMSD analysis of replica simulations: (a) IMPHY000687, (b) IMPHY004619, (c) IMPHY007084, (d) IMPHY012021, and (e) reference inhibitor; Figure S3: RMSF of apo-protein; Figure S4: RMSF analysis of protein replica simulations: (a) IMPHY000687, (b) IMPHY004619, (c) IMPHY007084, (d) IMPHY012021, and (e) reference inhibitor; Figure S5: RMSF analysis of ligand replica simulations: (a) IMPHY000687, (b) IMPHY004619, (c) IMPHY007084, (d) IMPHY012021, and (e) reference inhibitor; Figure S6: PCA of apo-protein; Figure S7: FEL of apo-protein; Figure S8: Global minimum conformations extracted from FEL analysis for HIV-RT complexes and used for structural superimposition. Panels (a1–a3) IMPHY000687, (b1–b3) IMPHY007084, (c1–c3) IMPHY004619, (d1–d3) IMPHY012021, and (e1–e3) reference inhibitor, showing representative low-energy binding poses adopted during the MD simulations; Figure S9: Detailed 2D interaction profiles of the selected reference inhibitors delavirdine, efavirenz, and nevirapine; Figure S10: Applicability domain analysis.
