# Supplementary material for: Anti-HIV Potential of Origanum vulgare Compounds Targeting Viral Reverse Transcriptase with High Binding and Stability Validated by Machine Learning
Source: Viruses. 2026 Sep 13;18(9):1010. doi: 10.3390/v18091010 (PMC13611907; doi:10.3390/v18091010)
Supplement: Supplementary file 1 [file viruses-18-01010-s001.zip › viruses-4504627-supplementary.pdf]

Supplementary File

| Compounds | Docking Score |
|---|---|
| IMPHY000687 | -9.429379238 |
| IMPHY007084 | -9.321850892 |
| IMPHY004619 | -9.246894315 |
| IMPHY012021 | -9.237496894 |
| IMPHY007172 | -9.165601787 |
| IMPHY004388 | -9.019179422 |
| IMPHY001838 | -9.012292143 |
| IMPHY004660 | -8.912149389 |
| IMPHY015047 | -8.905662352 |
| IMPHY004038 | -8.807398799 |
| IMPHY014925 | -8.746162944 |
| IMPHY000911 | -8.472932901 |
| IMPHY003325 | -8.445789293 |
| IMPHY017821 | -8.426987385 |
| IMPHY011933 | -8.377595471 |
| IMPHY011967 | -8.336882983 |
| IMPHY005431 | -8.332224706 |
| IMPHY012914 | -8.327340376 |
| IMPHY011844 | -8.249936537 |
| IMPHY004597 | -8.217294405 |
| IMPHY002073 | -8.215861401 |
| IMPHY015549 | -8.183345914 |
| IMPHY001246 | -8.077000463 |
| IMPHY011646 | -8.075381881 |
| IMPHY011371 | -8.028526867 |
| IMPHY016027 | -8.01002365 |
| IMPHY002072 | -7.931816227 |
| IMPHY012255 | -7.924990777 |
| IMPHY014732 | -7.913650544 |
| IMPHY006719 | -7.904792982 |
| IMPHY007526 | -7.895242855 |
| IMPHY015341 | -7.889373217 |
| IMPHY007201 | -7.874225339 |
| IMPHY012949 | -7.867309742 |
| IMPHY001931 | -7.85691101 |
| IMPHY012550 | -7.856286714 |
| IMPHY005843 | -7.847411486 |
| IMPHY013133 | -7.840683825 |
| IMPHY016397 | -7.840339665 |
| IMPHY017937 | -7.817267541 |
| IMPHY008707 | -7.810166247 |
| IMPHY004194 | -7.806037186 |
| IMPHY015095 | -7.800500734 |
| IMPHY011826 | -7.789532267 |

| | |
|---|---|
| IMPHY004281 | -7.757976405 |
| IMPHY011709 | -7.740692882 |
| IMPHY012292 | -7.727439331 |
| IMPHY011396 | -7.716776621 |
| IMPHY001817 | -7.693865972 |
| IMPHY011938 | -7.670351405 |
| IMPHY004661 | -7.668204985 |
| IMPHY014690 | -7.653000218 |
| IMPHY012265 | -7.647608709 |
| IMPHY009773 | -7.647543642 |
| IMPHY012916 | -7.643161326 |
| IMPHY010317 | -7.62308468 |
| IMPHY013574 | -7.622770171 |
| IMPHY011880 | -7.616969377 |
| IMPHY001218 | -7.591971217 |
| IMPHY013765 | -7.553112562 |
| IMPHY015040 | -7.546070526 |
| IMPHY006325 | -7.505721809 |
| IMPHY016014 | -7.493778838 |
| IMPHY014906 | -7.481300039 |
| IMPHY006550 | -7.475789146 |
| IMPHY011066 | -7.475572413 |
| IMPHY014847 | -7.448557174 |
| IMPHY016473 | -7.439461099 |
| IMPHY004077 | -7.437902832 |
| IMPHY012278 | -7.435505341 |
| IMPHY004433 | -7.4293997 |
| IMPHY009740 | -7.421986594 |
| IMPHY015570 | -7.420388404 |
| IMPHY006696 | -7.413806833 |
| IMPHY013764 | -7.408423202 |
| IMPHY010550 | -7.407029179 |
| IMPHY011354 | -7.406393723 |
| IMPHY012290 | -7.396166496 |
| IMPHY011342 | -7.375302582 |
| IMPHY001854 | -7.356417277 |
| IMPHY011187 | -7.348842741 |
| IMPHY012152 | -7.347772092 |
| IMPHY012036 | -7.335865202 |
| IMPHY012157 | -7.334460675 |
| IMPHY011590 | -7.333918775 |
| IMPHY012202 | -7.329374233 |
| IMPHY016053 | -7.326571736 |
| IMPHY011901 | -7.311270741 |
| IMPHY009946 | -7.303768199 |
| IMPHY006965 | -7.299138346 |

| | |
|---|---|
| IMPHY009739 | -7.294957991 |
| IMPHY000099 | -7.289765186 |
| IMPHY012920 | -7.286388304 |
| IMPHY012178 | -7.283673006 |
| IMPHY011939 | -7.280574663 |
| IMPHY015042 | -7.277091157 |
| IMPHY006485 | -7.273565406 |
| IMPHY003536 | -7.263482786 |
| IMPHY009758 | -7.255625807 |
| IMPHY009268 | -7.252941422 |
| IMPHY009752 | -7.252209733 |
| IMPHY009874 | -7.249263442 |
| IMPHY016840 | -7.233739754 |
| IMPHY015016 | -7.226820743 |
| IMPHY001816 | -7.224955496 |
| IMPHY014094 | -7.206217625 |
| IMPHY016409 | -7.201400053 |
| IMPHY003296 | -7.195146174 |
| IMPHY011542 | -7.183722289 |
| IMPHY000915 | -7.179103732 |
| IMPHY015004 | -7.173691021 |
| IMPHY016322 | -7.172224297 |
| IMPHY014914 | -7.138621046 |
| IMPHY010887 | -7.132675519 |
| IMPHY011973 | -7.130473213 |
| IMPHY012586 | -7.117243781 |
| IMPHY011988 | -7.098200263 |
| IMPHY011557 | -7.094769993 |
| IMPHY014989 | -7.0934474 |
| IMPHY011884 | -7.091024737 |
| IMPHY012585 | -7.082049442 |
| IMPHY002103 | -7.076465195 |
| IMPHY011902 | -7.073238689 |
| IMPHY014836 | -7.043784218 |
| IMPHY015003 | -7.043734233 |
| IMPHY010049 | -7.035049324 |
| IMPHY003602 | -7.033471388 |
| IMPHY015098 | -7.016014718 |
| IMPHY016514 | -7.012325774 |
| IMPHY011519 | -6.9903613 |
| IMPHY016466 | -6.985856225 |
| IMPHY013971 | -6.975230853 |
| IMPHY012256 | -6.965410932 |
| IMPHY014873 | -6.965410932 |
| IMPHY004003 | -6.957311805 |
| IMPHY012002 | -6.941541146 |

| | |
|---|---|
| IMPHY012072 | -6.928386661 |
| IMPHY004049 | -6.904468249 |
| IMPHY011532 | -6.899158418 |
| IMPHY002926 | -6.891141431 |
| IMPHY003486 | -6.883875387 |
| IMPHY012075 | -6.872362575 |
| IMPHY010097 | -6.839615755 |
| IMPHY014877 | -6.822113315 |
| IMPHY007840 | -6.812794541 |
| IMPHY007747 | -6.805959379 |
| IMPHY003398 | -6.788404463 |
| IMPHY012160 | -6.784150229 |
| IMPHY006138 | -6.755725146 |
| IMPHY011872 | -6.746286778 |
| IMPHY006404 | -6.733454801 |
| IMPHY013577 | -6.712628264 |
| IMPHY011890 | -6.7037373 |
| IMPHY012048 | -6.702971096 |
| IMPHY012915 | -6.678446351 |
| IMPHY003710 | -6.639979446 |
| IMPHY004542 | -6.613185099 |
| IMPHY011648 | -6.612804602 |
| IMPHY002825 | -6.599198683 |
| IMPHY006660 | -6.572873575 |
| IMPHY006948 | -6.564817413 |
| IMPHY016898 | -6.556202479 |
| IMPHY011647 | -6.553195827 |
| IMPHY011749 | -6.529613826 |
| IMPHY016742 | -6.506048733 |
| IMPHY014986 | -6.490152488 |
| IMPHY011762 | -6.489367354 |
| IMPHY011381 | -6.471001384 |
| IMPHY017655 | -6.469206644 |
| IMPHY011763 | -6.465789533 |
| IMPHY012487 | -6.463407756 |
| IMPHY012667 | -6.444364774 |
| IMPHY015435 | -6.416083067 |
| IMPHY015646 | -6.407687449 |
| IMPHY001658 | -6.350829196 |
| IMPHY012665 | -6.31044595 |
| IMPHY008024 | -6.230954881 |
| IMPHY009863 | -6.206552196 |
| IMPHY009846 | -6.186114535 |
| IMPHY007067 | -6.082051925 |
| IMPHY007317 | -6.048830236 |
| IMPHY006944 | -6.041656785 |

| | |
|---|---|
| IMPHY007331 | -6.03253205 |
| IMPHY003513 | -6.010322091 |
| IMPHY006419 | -5.92940658 |
| IMPHY005390 | -5.878572597 |
| IMPHY010781 | -5.865063449 |
| IMPHY001548 | -5.851912668 |
| IMPHY004541 | -5.847181292 |
| IMPHY011960 | -5.80342708 |
| IMPHY014907 | -5.802731749 |
| IMPHY012086 | -5.793932053 |
| IMPHY009832 | -5.788911112 |
| IMPHY011789 | -5.763499325 |
| IMPHY009964 | -5.704131355 |
| IMPHY006032 | -5.62945508 |
| IMPHY001555 | -5.602580856 |
| IMPHY012104 | -5.57051349 |
| IMPHY004121 | -5.557457647 |
| IMPHY011790 | -5.550926998 |
| IMPHY012654 | -5.521876501 |
| IMPHY013799 | -5.506138478 |
| IMPHY011694 | -5.481314233 |
| IMPHY014882 | -5.41947306 |
| IMPHY016363 | -5.370285955 |
| IMPHY012058 | -5.310711501 |
| IMPHY014923 | -5.297451205 |
| IMPHY003179 | -5.197822383 |
| IMPHY001828 | -5.065297736 |
| IMPHY016015 | -5.062440828 |
| IMPHY006347 | -5.015986473 |
| IMPHY006661 | -4.987118315 |
| IMPHY011562 | -4.963803503 |
| IMPHY000573 | -4.94395443 |
| IMPHY006353 | -4.835697619 |
| IMPHY003720 | -4.787065953 |
| IMPHY012168 | -4.760754898 |
| IMPHY015123 | -4.760754898 |
| IMPHY007131 | -4.709476894 |
| IMPHY003759 | -4.695284205 |
| IMPHY006526 | -4.64357516 |
| IMPHY009737 | -4.62568925 |
| IMPHY012268 | -4.601673123 |
| IMPHY003760 | -4.51480786 |
| IMPHY015128 | -4.477048463 |
| IMPHY009871 | -4.475701763 |
| IMPHY003525 | -4.439692561 |
| IMPHY005345 | -4.423363896 |

| | |
|---|---|
| IMPHY007852 | -4.423034435 |
| IMPHY000602 | -4.370582728 |
| IMPHY003132 | -4.365041706 |
| IMPHY003977 | -4.363475881 |
| IMPHY013080 | -4.349697826 |
| IMPHY009729 | -4.341660258 |
| IMPHY012100 | -4.330855776 |
| IMPHY009838 | -4.291155006 |
| IMPHY011896 | -4.289567523 |
| IMPHY003616 | -4.269584207 |
| IMPHY011320 | -4.266660464 |
| IMPHY012738 | -4.264690435 |
| IMPHY003982 | -4.260461443 |
| IMPHY014811 | -4.244810675 |
| IMPHY015633 | -4.243783743 |
| IMPHY003719 | -4.243739801 |
| IMPHY014831 | -4.204241573 |
| IMPHY010072 | -4.201246131 |
| IMPHY014852 | -4.188103138 |
| IMPHY001135 | -4.182106818 |
| IMPHY006952 | -4.180698272 |
| IMPHY016020 | -4.173014701 |
| IMPHY012192 | -4.14828164 |
| IMPHY006414 | -4.142521784 |
| IMPHY011745 | -4.115855349 |
| IMPHY004680 | -4.107107607 |
| IMPHY007520 | -4.096521071 |
| IMPHY011659 | -4.089086522 |
| IMPHY013572 | -4.068378897 |
| IMPHY014870 | -4.066544969 |
| IMPHY014871 | -4.05154594 |
| IMPHY012147 | -4.048527436 |
| IMPHY015629 | -4.039029343 |
| IMPHY006145 | -4.00385552 |
| IMPHY011965 | -3.982659685 |
| IMPHY014865 | -3.975921966 |
| IMPHY011632 | -3.97416861 |
| IMPHY000491 | -3.965618051 |
| IMPHY011599 | -3.964301878 |
| IMPHY009853 | -3.951609435 |
| IMPHY009778 | -3.942546099 |
| IMPHY011643 | -3.942198682 |
| IMPHY011063 | -3.928169061 |
| IMPHY009743 | -3.925392808 |
| IMPHY003567 | -3.920361943 |
| IMPHY011069 | -3.892732462 |

| | |
|---|---|
| IMPHY003694 | -3.886481505 |
| IMPHY011630 | -3.869126011 |
| IMPHY009765 | -3.862688301 |
| IMPHY012061 | -3.838979236 |
| IMPHY011660 | -3.83248041 |
| IMPHY014708 | -3.820826264 |
| IMPHY012261 | -3.815213051 |
| IMPHY011761 | -3.812092154 |
| IMPHY006070 | -3.810319704 |
| IMPHY011793 | -3.807852658 |
| IMPHY012165 | -3.798866452 |
| IMPHY014806 | -3.777046007 |
| IMPHY011552 | -3.767818427 |
| IMPHY011564 | -3.767666912 |
| IMPHY012910 | -3.732133923 |
| IMPHY015022 | -3.718987729 |
| IMPHY010609 | -3.714817895 |
| IMPHY011633 | -3.711288537 |
| IMPHY011792 | -3.71121368 |
| IMPHY007376 | -3.708754788 |
| IMPHY011957 | -3.70171602 |
| IMPHY011536 | -3.701600821 |
| IMPHY014863 | -3.695657978 |
| IMPHY011004 | -3.690863668 |
| IMPHY011586 | -3.672712056 |
| IMPHY016012 | -3.655826638 |
| IMPHY012912 | -3.629774019 |
| IMPHY016054 | -3.628967105 |
| IMPHY006324 | -3.592850027 |
| IMPHY014801 | -3.591610689 |
| IMPHY014874 | -3.554650784 |
| IMPHY014833 | -3.542365792 |
| IMPHY006950 | -3.531320309 |
| IMPHY015799 | -3.512747726 |
| IMPHY011392 | -3.491157511 |
| IMPHY014958 | -3.48504228 |
| IMPHY005940 | -3.483068466 |
| IMPHY007421 | -3.477415673 |
| IMPHY012095 | -3.473544765 |
| IMPHY016866 | -3.470935819 |
| IMPHY012921 | -3.429737141 |
| IMPHY008150 | -3.418287386 |
| IMPHY003695 | -3.392737287 |
| IMPHY014240 | -3.38138462 |
| IMPHY014817 | -3.375968077 |
| IMPHY007307 | -3.325290031 |

| | |
|---|---|
| IMPHY011667 | -3.31270427 |
| IMPHY013093 | -3.309087647 |
| IMPHY014988 | -3.290076937 |
| IMPHY012589 | -3.25669965 |
| IMPHY006417 | -3.229686889 |
| IMPHY011838 | -3.175734937 |
| IMPHY003807 | -3.158656911 |
| IMPHY013937 | -3.147236784 |
| IMPHY012175 | -3.141237513 |
| IMPHY000399 | -3.074043933 |
| IMPHY010080 | -3.046727045 |
| IMPHY006007 | -3.002244593 |
| IMPHY012712 | -2.772623093 |
| IMPHY012093 | -2.616015455 |
| IMPHY012739 | -2.43783432 |
| IMPHY011521 | -2.287664297 |
| IMPHY003518 | -2.235868316 |
| IMPHY014835 | -2.227128495 |
| IMPHY014115 | -2.20992249 |
| IMPHY011797 | -2.04130382 |
| IMPHY012723 | -2.038621256 |
| IMPHY004669 | -1.970179604 |
| IMPHY004631 | -1.854085004 |
| IMPHY007327 | -1.848785065 |
| IMPHY014990 | -1.835944777 |
| IMPHY001516 | -1.362791888 |
| IMPHY007202 | -1.341168206 |
| IMPHY009490 | -1.28578839 |
| IMPHY003485 | -1.254647176 |
| IMPHY006970 | -1.225526865 |
| IMPHY007204 | -1.066870003 |
| IMPHY011817 | -1.030029984 |
| IMPHY003104 | -1.014460408 |

Supplementary Table S1. The list compounds along with docking score after virtual screening.

| Name | Leverage | Leverage_AD | Distance_Cutoff_95th | Distance_AD | Final_Applicability_Domain |
|---|---|---|---|---|---|
| IMPHY007084 | -64242362563 | Inside AD | 26.82 | Inside AD | Inside AD |
| IMPHY012021 | -7.08206E+11 | Inside AD | 26.82 | Inside AD | Inside AD |
| IMPHY000687 | 3.53321E+11 | Outside AD | 26.82 | Inside AD | Outside AD |
| Reference | -1.53152E+12 | Inside AD | 26.82 | Inside AD | Inside AD |
| IMPHY004619 | 1.52687E+12 | Outside AD | 26.82 | Inside AD | Outside AD |

Supplementary Table S2. Leverage- and distance-based applicability domain (AD) analysis indicating the prediction reliability of the shortlisted phytochemicals and the reference inhibitor.

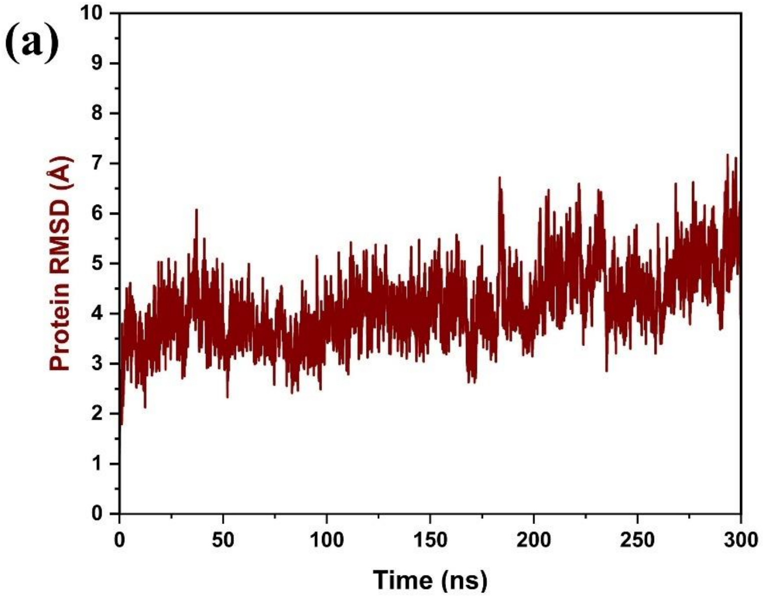


Supplementary Figure S1. RMSD of Apo-protein

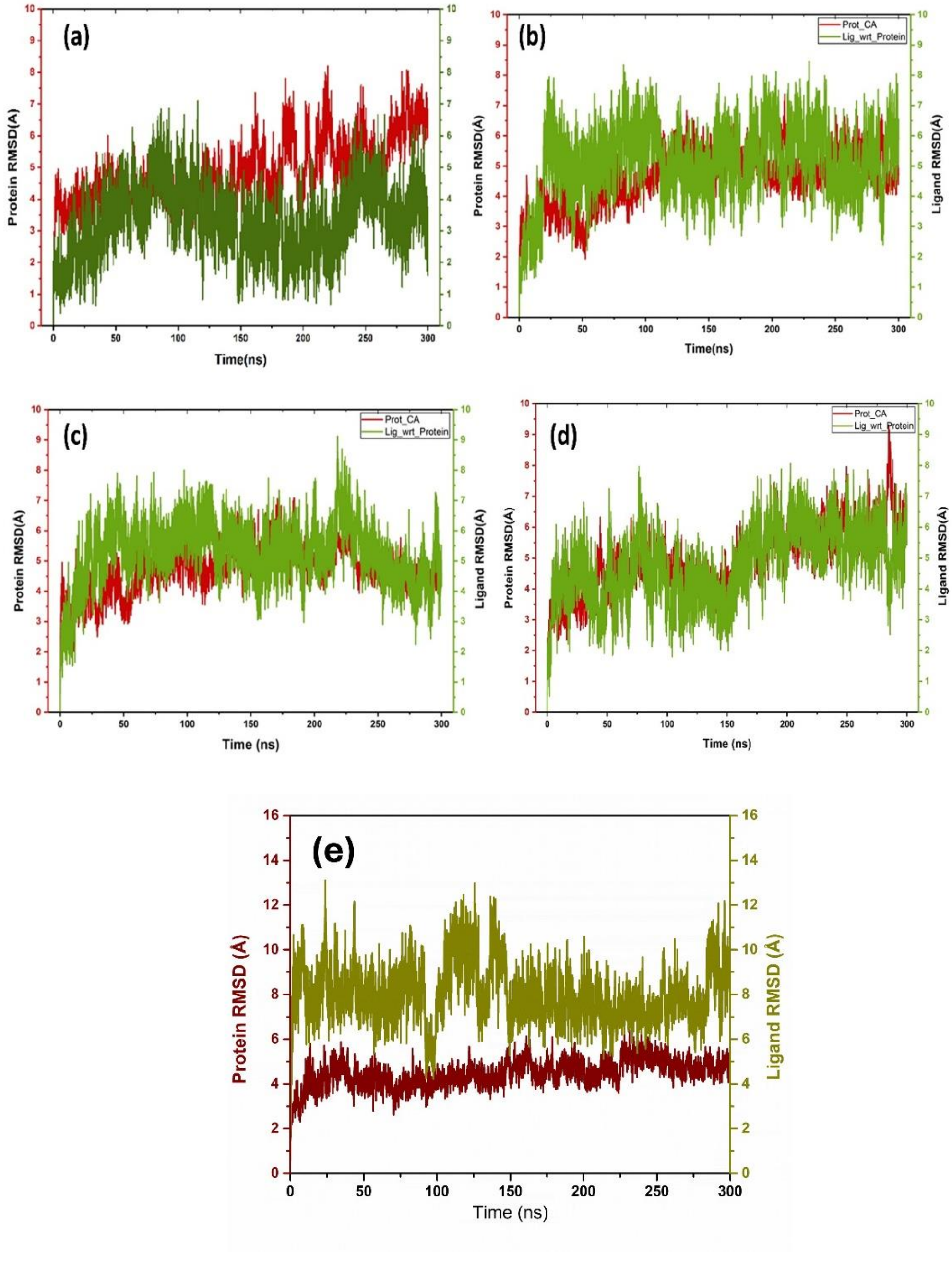


Supplementary Figure S2: RMSD analysis of replica simulation (a) IMPHY000687, (b) IMPHY004619, (c) IMPHY007084, (d) IMPHY012021, (e) Reference inhibitor

(a)

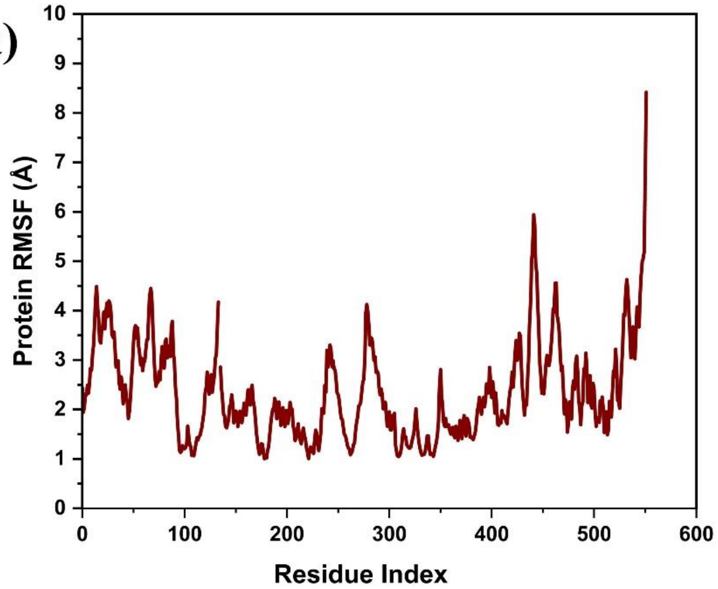


Supplementary Figure S3. RMSF of Apo-protein

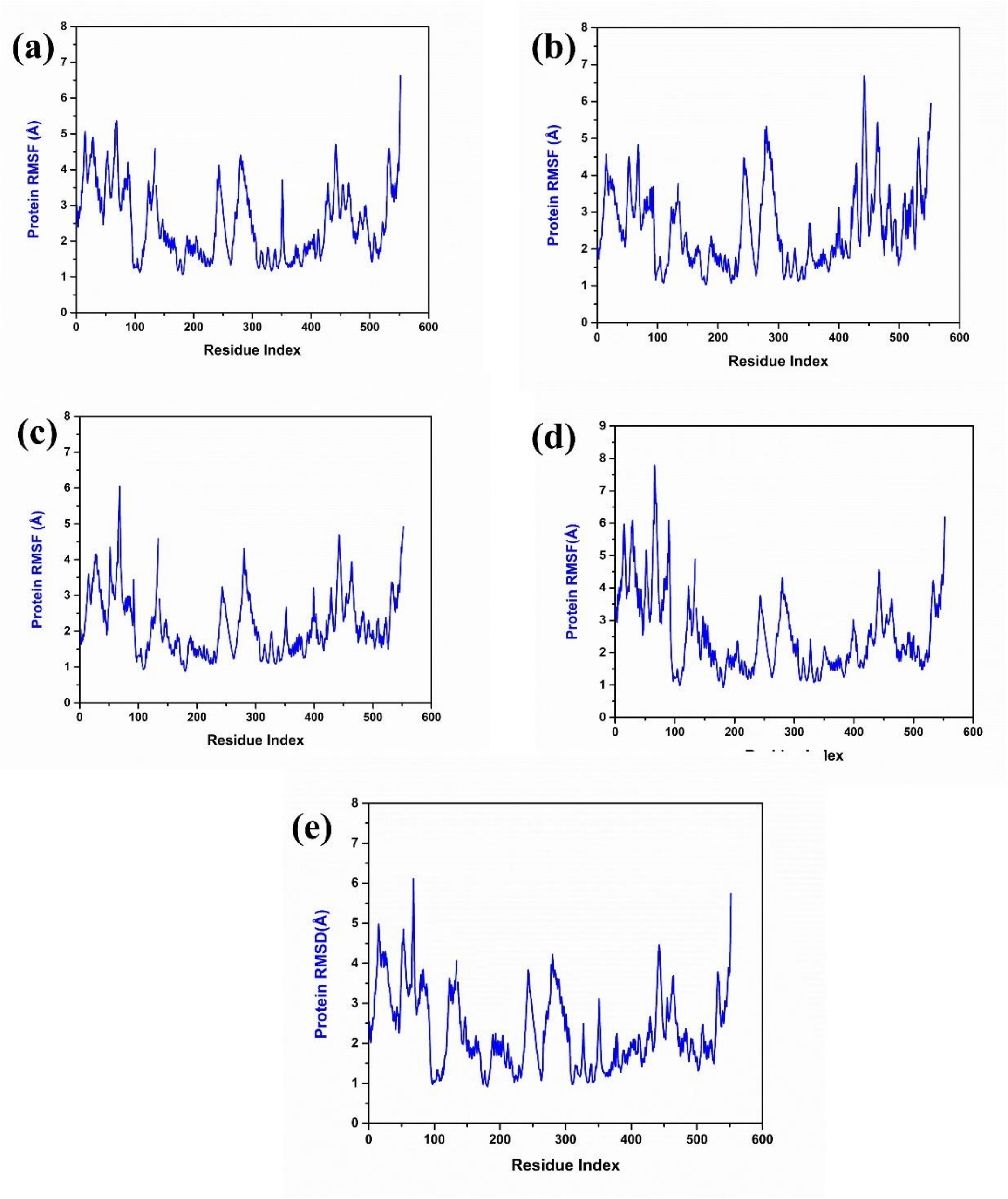


Supplementary Figure S4: RMSF analysis of protein replica simulation (a) IMPHY000687, (b) IMPHY004619, (c), IMPHY007084, (d) IMPHY012021 (e) Reference inhibitor

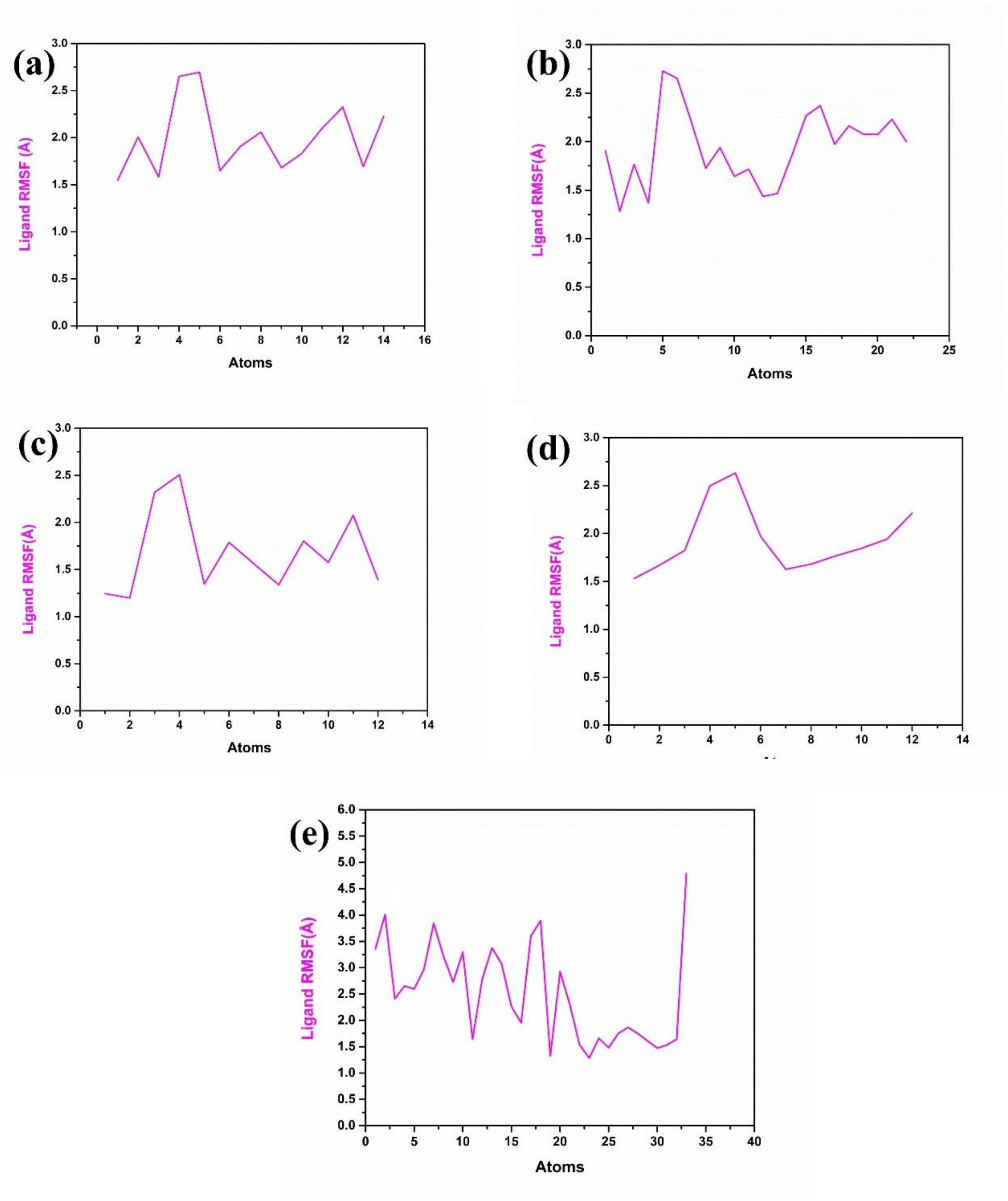


Supplementary Figure S5: RMSF analysis of Ligand replica simulation (a) IMPHY000687, (b) IMPHY004619, (c), IMPHY007084, (d) IMPHY012021 (e) Reference inhibitor

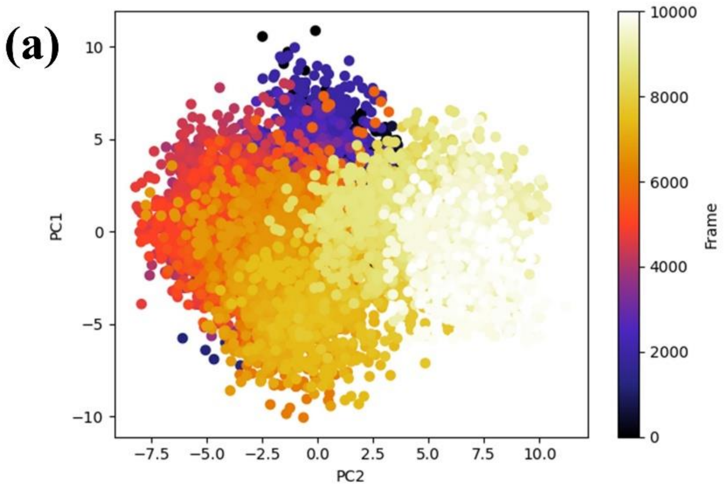


Supplementary Figure S6. PCA of Apo-protein

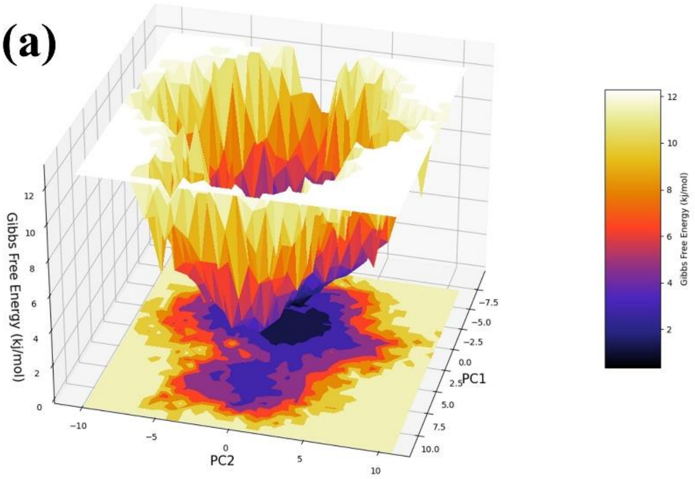


Supplementary Figure S7. FEL of apo-protein

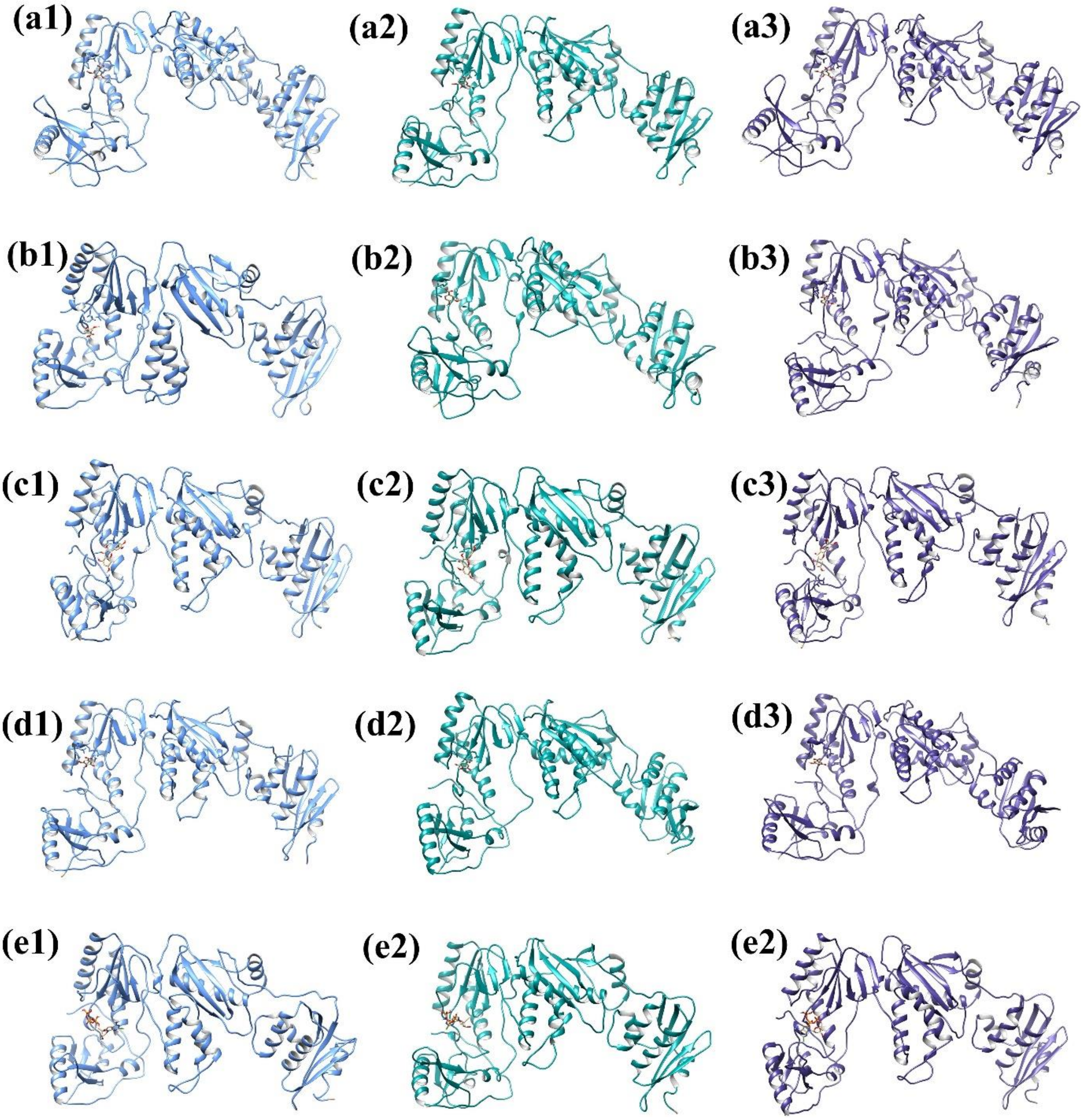


Supplementary Figure S8. Global minimum conformations extracted from FEL analysis for HIV-RT complexes and used for structural superimposition. Panels (a1–a3) IMPHY000687, (b1–b3) IMPHY007084, (c1–c3) IMPHY004619, (d1–d3) IMPHY012021, and (e1–e3) reference inhibitor, showing representative low-energy binding poses adopted during the MD simulations

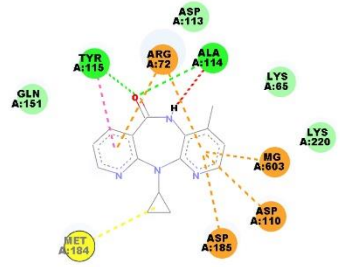


Nevirapine

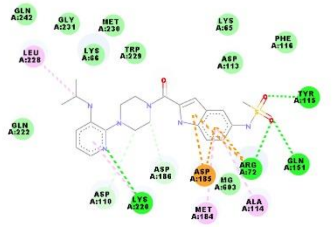


Delavirdine

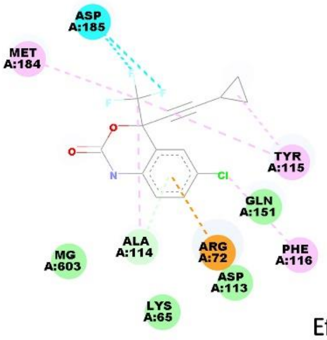


Efavirenz

Supplementary Figure S9: detailed 2D interaction profiles of the selected reference inhibitors delavirdine, efavirenz, and nevirapine.

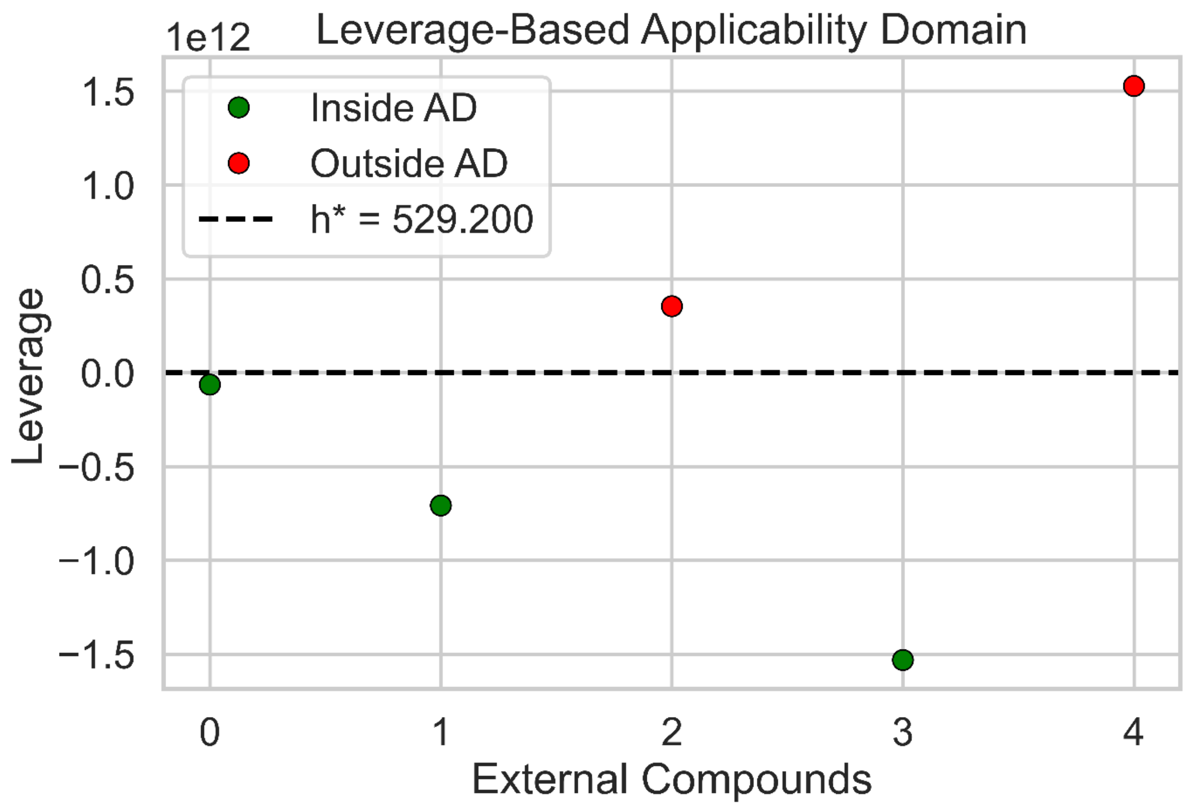


Supplementary Figure S10 – Applicability domain Analysis
